# Supplementary figures and images for: The Rheb GTPase promotes pheromone blindness via a TORC1-independent pathway in the phytopathogenic fungus Ustilago maydis
Source: PLoS Genet. 2022 Nov 14;18(11):e1010483. doi: 10.1371/journal.pgen.1010483 (PMC9704768; doi:10.1371/journal.pgen.1010483)

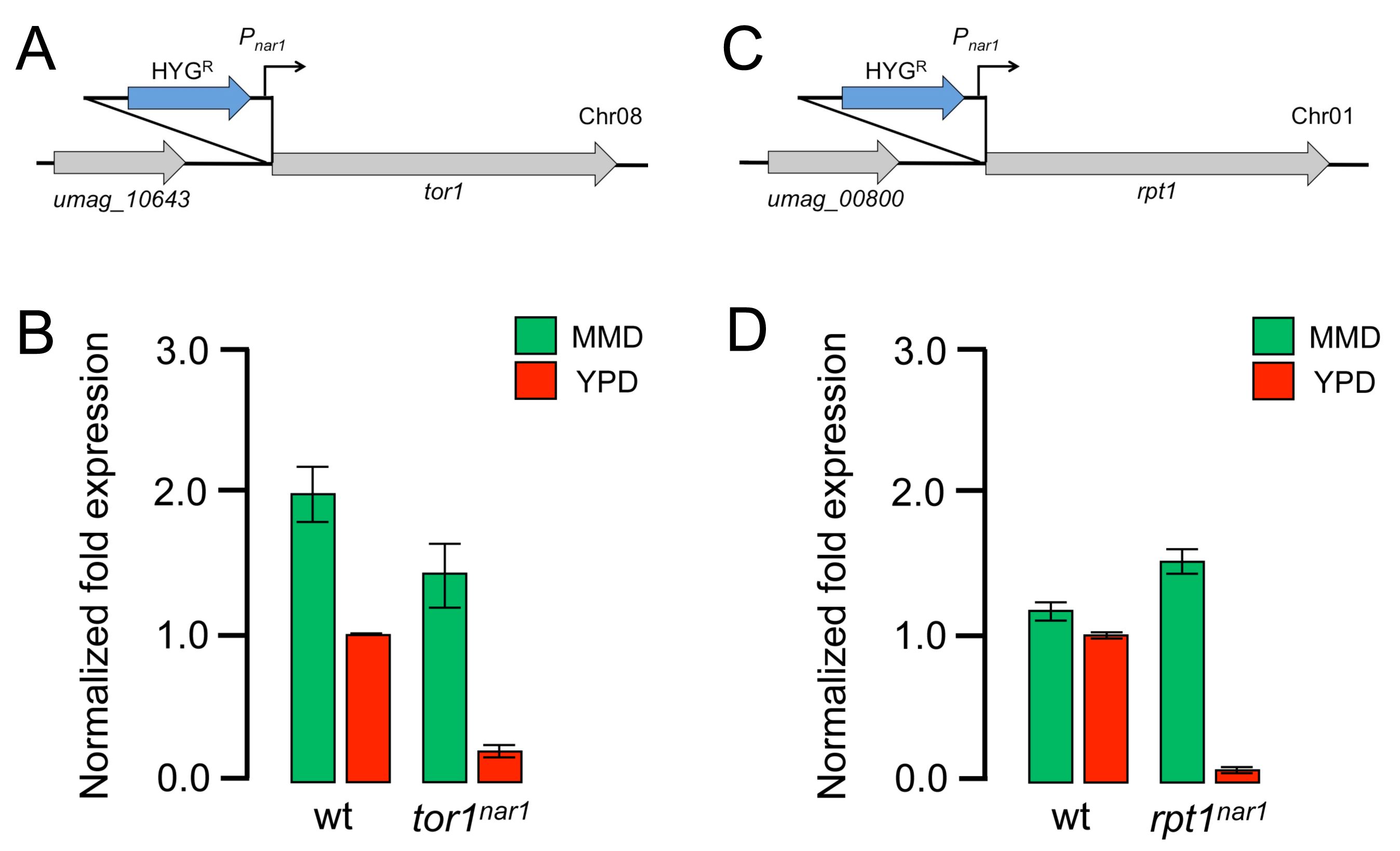

Supplement: S1 Fig — (A and C) schemes of tor1nar1 and rpt1nar1 conditional alleles. (B and D) qRT-PCR for tor1 and rpt1 mRNA levels from control and tor1nar1 or rpt1nar1 conditional cells, incubated for 8 hours in YPD (repressive conditions) or in nitrate minimal medium (MMD, permissive conditions). Values are referred to the expression of tor1 or rpt1 in FB1 (control strain) grown in YPD. Each column represents the mean value of three independent biological replicates. Error bars represent the SD. (TIF) [file pgen.1010483.s001.tif]

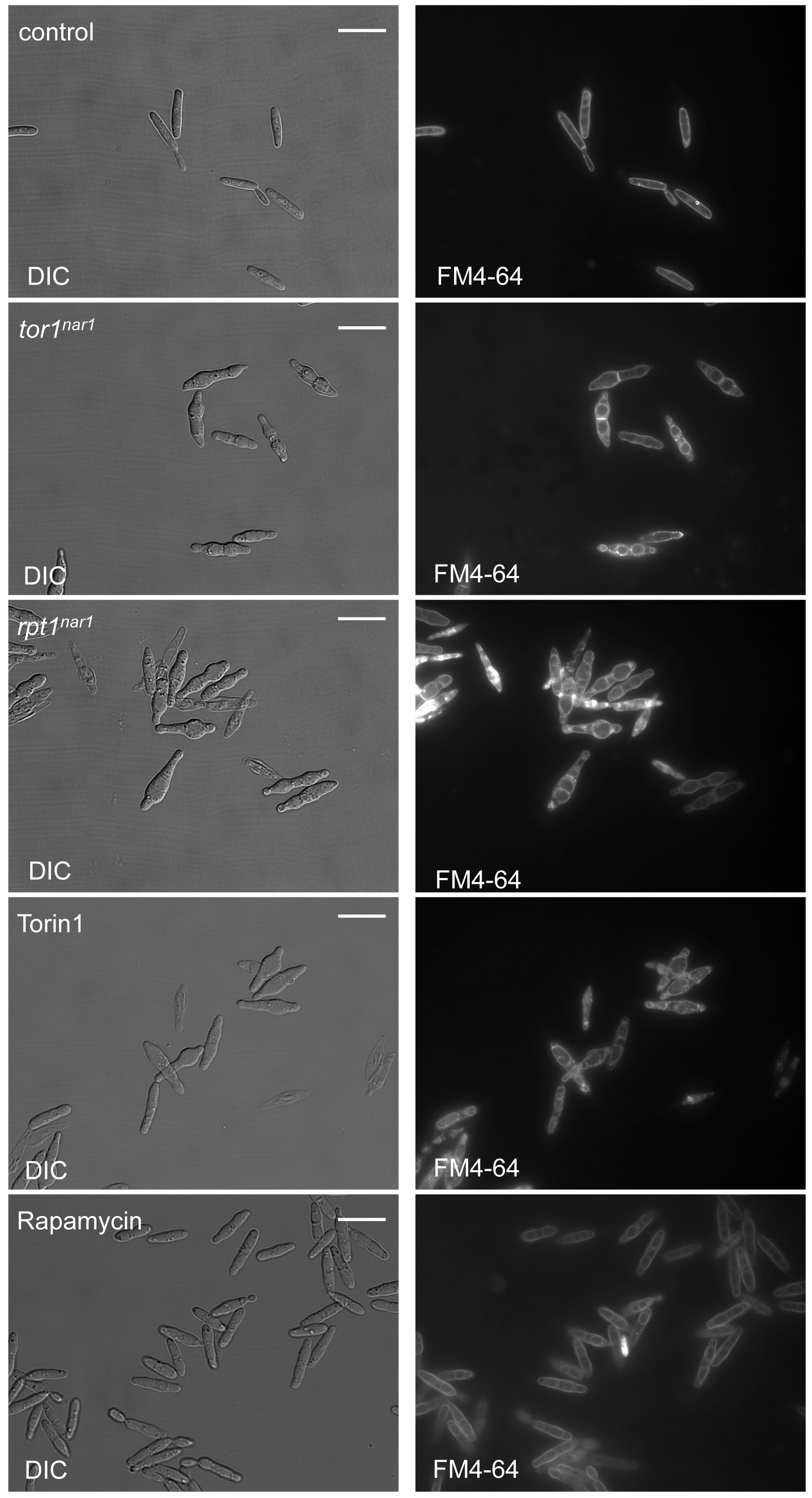

Supplement: S2 Fig — Representative images of liquid cultures of cells carrying the indicated conditional allele or treated with 10 μM Torin1 and 1μg/ml rapamycin (control: FB1), incubated in YPD for 8 hours. Fluorescent images showed FM4-64 staining. Bar: 15 μm. (TIF) [file pgen.1010483.s002.tif]

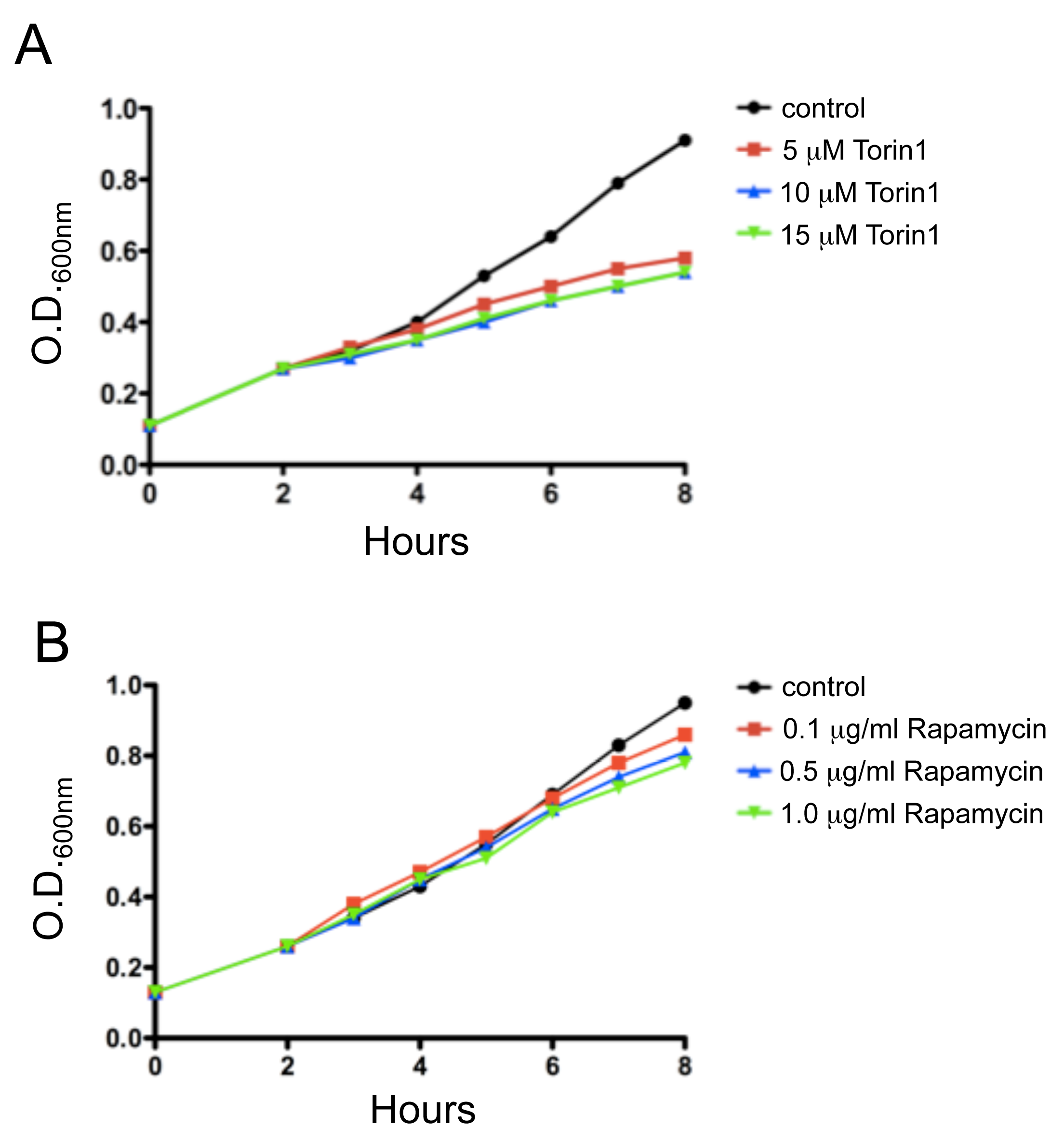

Supplement: S3 Fig — Growth curves of liquid YPD cultures of FB1 cells amended with the indicated concentrations of Torin1 or rapamycin. Control conditions included 1% DMSO as solvent. (TIF) [file pgen.1010483.s003.tif]

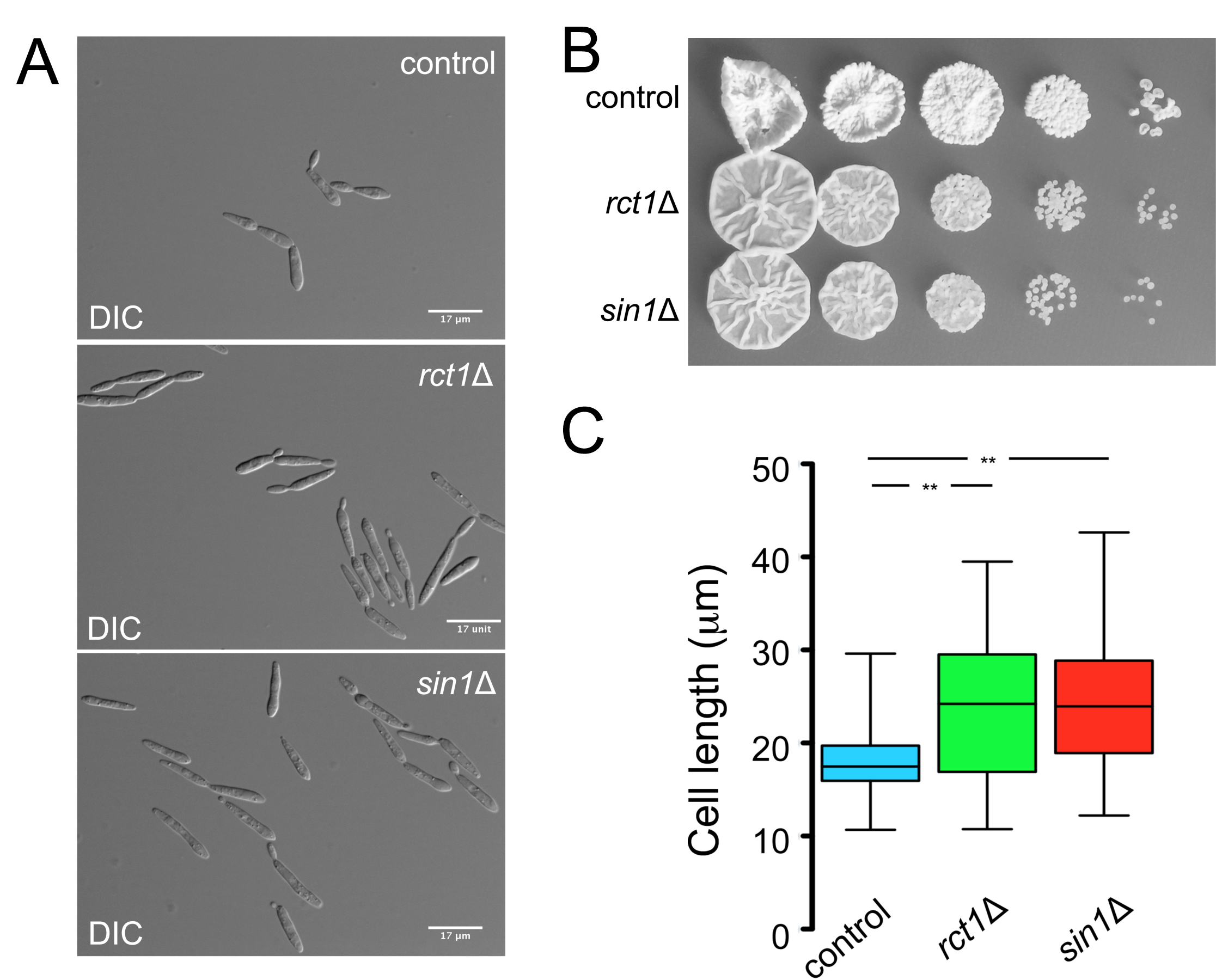

Supplement: S4 Fig — (A) Representative images of liquid cultures of cells carrying the indicated mutant allele incubated in YPD for 6 hours. Bar: 17 μm. (B) serial tenfold dilutions of cultures from strains carrying the indicated mutant alleles (control: FB1), spotted in solid YPD. Plates were incubated for 3 days at 28°. C, cell length of TORC2 mutant cells. The graph shows the result from three independent experiments, counting more than 50 cells. **p<0.01 based on a two-tailed Student´s t-test to control sample. (TIF) [file pgen.1010483.s004.tif]

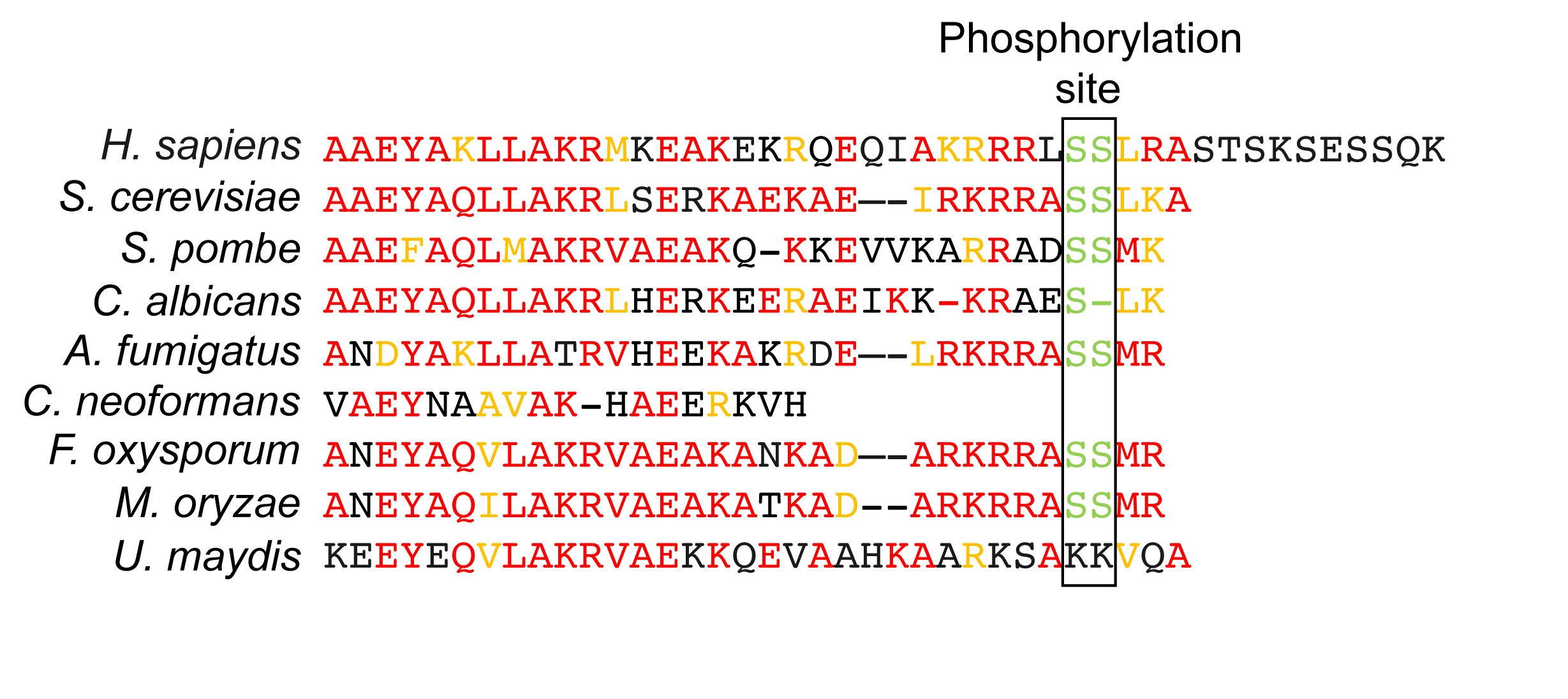

Supplement: S5 Fig — (TIF) [file pgen.1010483.s005.tif]

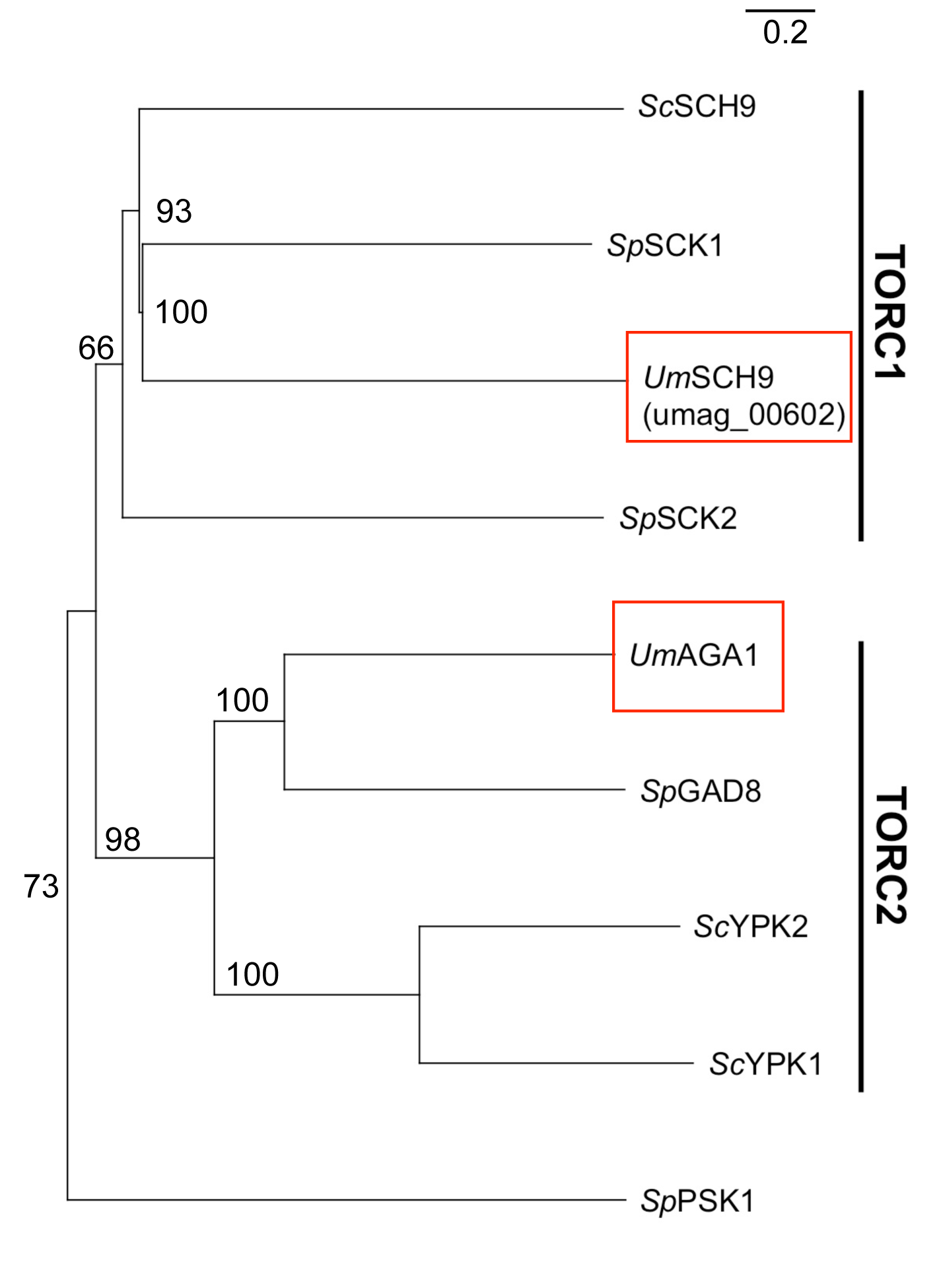

Supplement: S6 Fig — The tree was created by a neighbor-joining analysis without distance corrections using ClustalW. The scale bar denotes substitutions per site. The AGC association to TORC1 or TORC2 is indicated in the cases of budding and fission yeast. Note that in U. maydis, Aga1 was included in the group of TORC2 effectors, while Sch9 was included in the TORC1 effectors. (TIF) [file pgen.1010483.s006.tif]

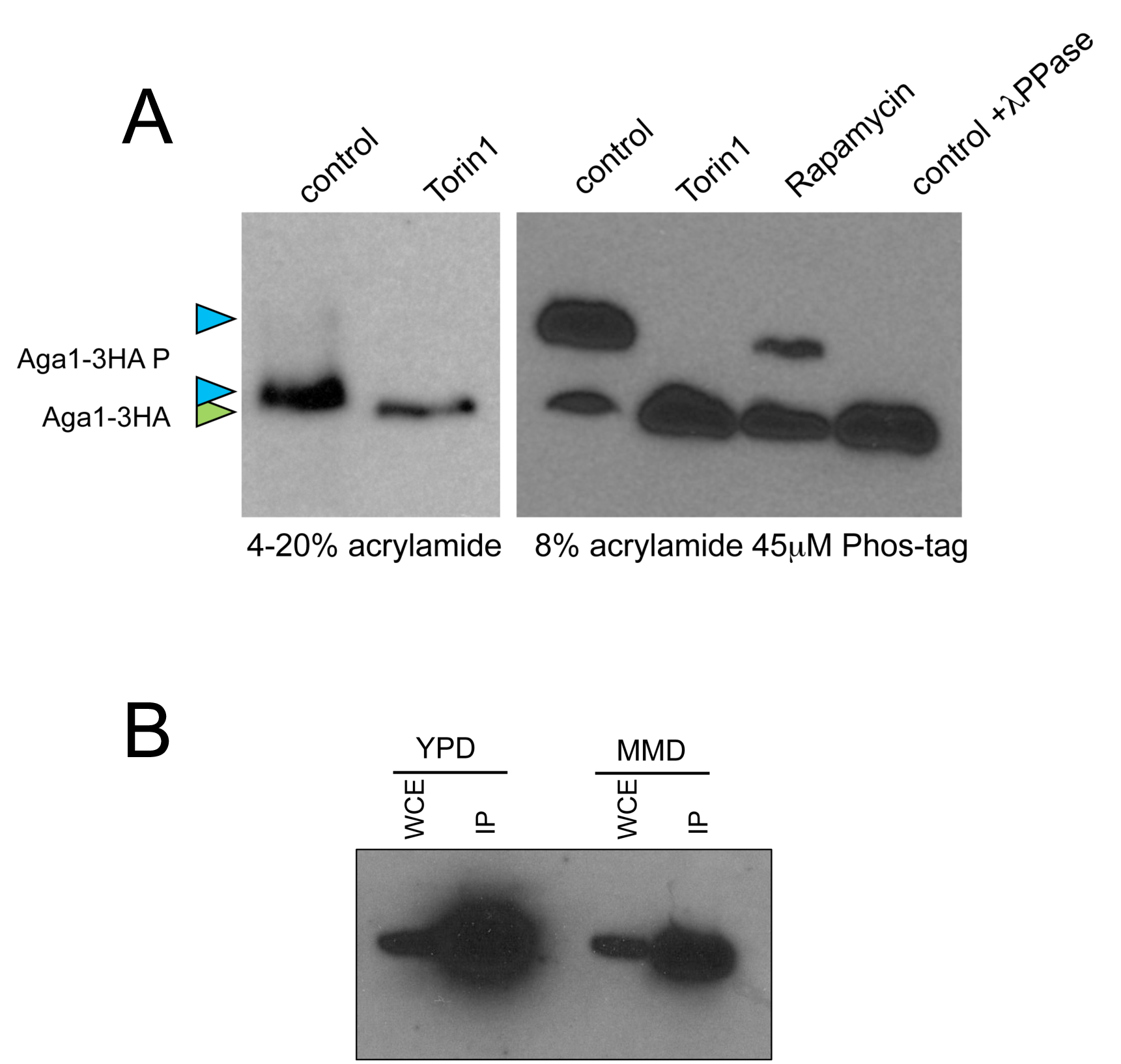

Supplement: S7 Fig — (A) Our first attempts to discriminate phosphorylated forms of Aga1-3HA were made using BioRad TGX (4–20% acrylamide) gels from TCA samples (left panel). Although a faint band shift was observed, it was not consistently reproduced. Using Phos-tag gels (right panel) improved the differential electrophoretic mobility. We have tried both TCA extracts and immunoprecipitated samples, and we have found that immunoprecipitated samples resulted in the more reproducible and consistent data. (B) Western blot of 5 microliters from crude extract (WCE) or immunoprecipitated (IP) samples extracted from cultures carrying the Aga1-HA allele grown on YPD or minimal medium amended with nitrate (MMD). The samples were loaded in BioRad TGX (4–20% acrylamide) gels. (TIF) [file pgen.1010483.s007.tif]

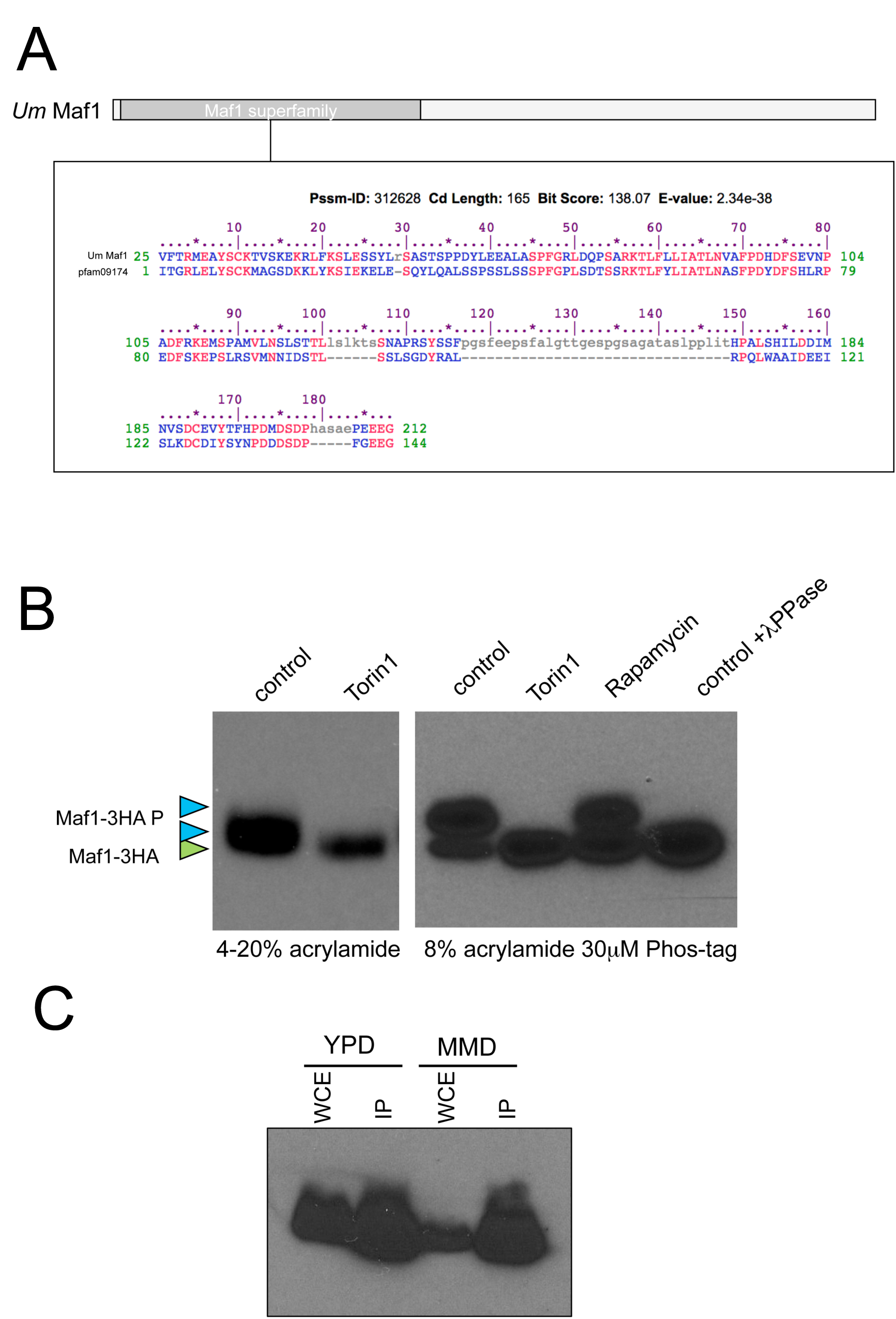

Supplement: S8 Fig — (A) Protein alignment showing the region of similarity between the predicted U. maydis Maf1 and the pfam indicative of Maf1 superfamily. (B) electrophoretic mobilities of Maf1-3HA samples separated in TGX (TCA samples) or Phos-tag gels (immunoprecipitated samples). (C) Western blot of 5 microliters from crude extract (WCE) or immunoprecipitated (IP) samples extracted from cultures carrying the Maf1-HA allele grown on YPD or minimal medium amended with nitrate (MMD). The samples were loaded in BioRad TGX (4–20% acrylamide) gels. (TIF) [file pgen.1010483.s008.tif]

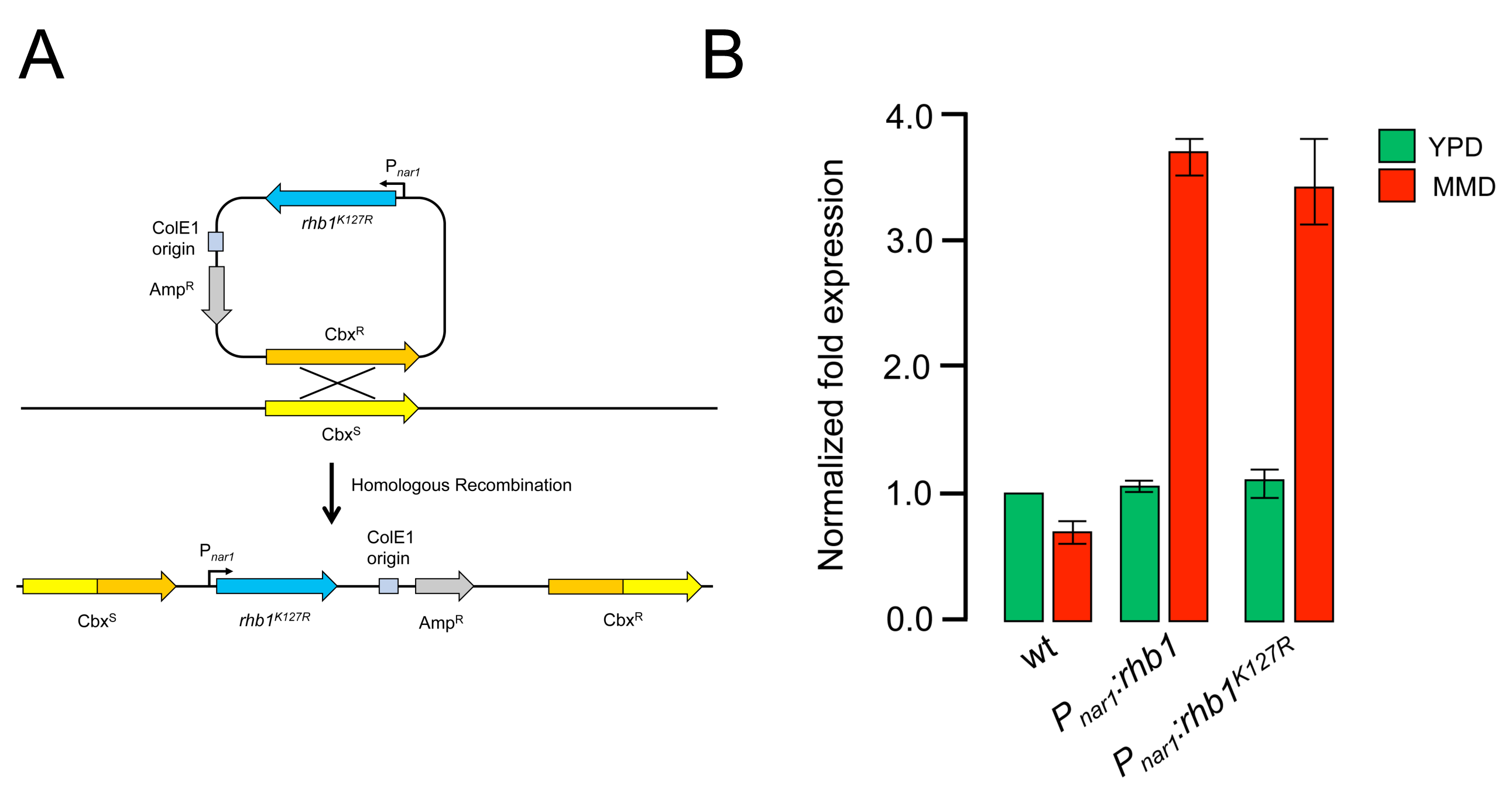

Supplement: S9 Fig — (A) Scheme for the integration of the plasmid harboring the ectopic rhb1K127R allele under nar1 promoter. Integration takes place at the ip locus (conferring resistance to carboxin). (B) qRT-PCR of rhb1 mRNA levels from control and strains carrying the indicated ectopic alleles, incubated for 8 hours in YPD (repressive conditions) or in nitrate minimal medium (MMD, permissive conditions). Values are referred to the expression of rhb1 in FB1 (control strain) grown in YPD. Each column represents the mean value of three independent biological replicates. Error bars represent the SD. (TIF) [file pgen.1010483.s009.tif]

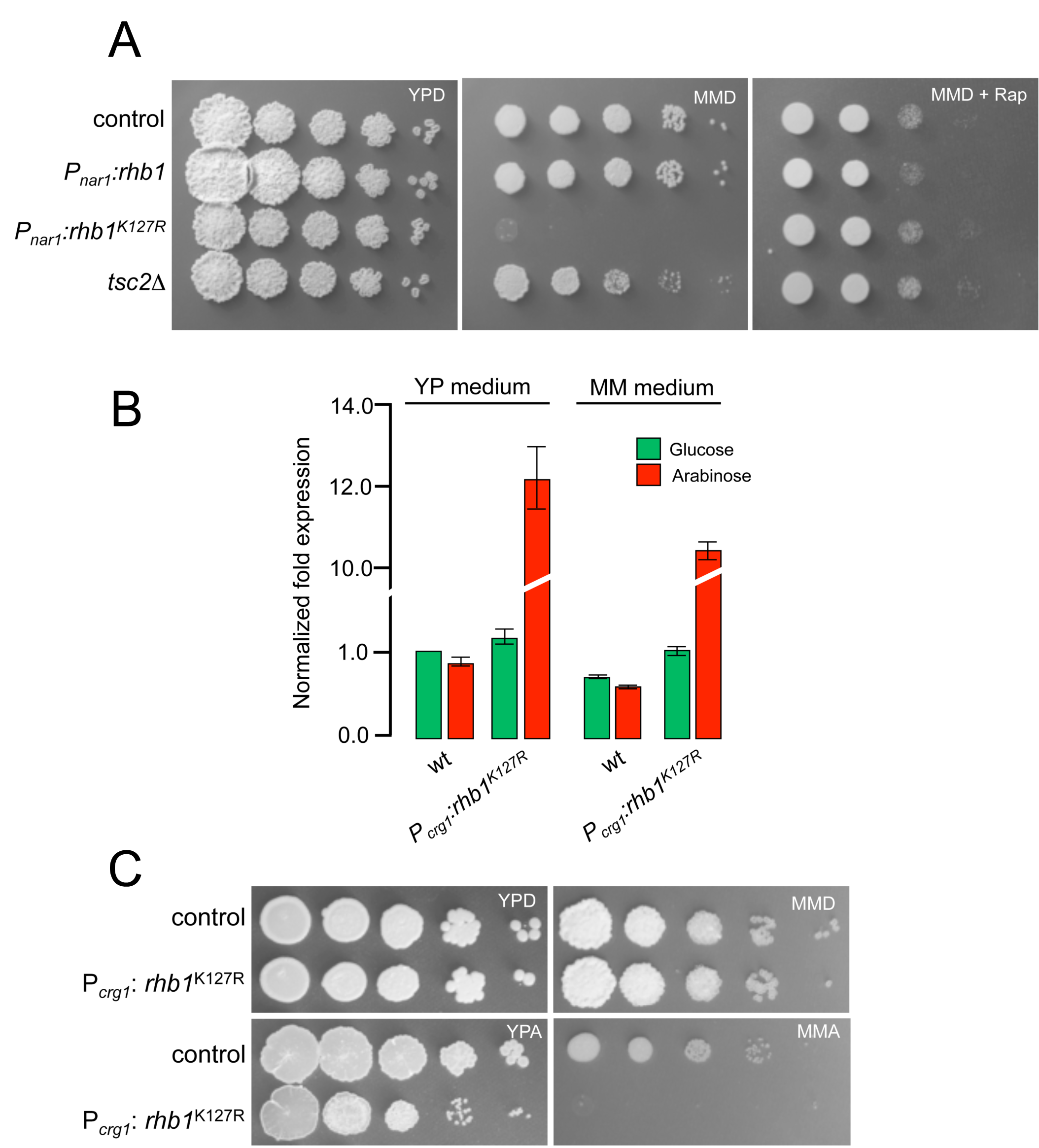

Supplement: S10 Fig — (A) Cells carrying mutations that upregulated Rheb were affected in growth in minimal medium amended with nitrate. Serial tenfold dilutions of cultures from the indicated strains (control: FB1), spotted in solid yeast-peptone medium (YPD, restrictive conditions for Pnar1 expression) or solid nitrate minimal medium (MMD, permissive conditions for Pnar1 expression), with or without 1μg/ml rapamycin. Plates were incubated for 3 days at 28°C. (B) Ectopic expression of rhb1K127R allele under crg1 promoter. qRT-PCR of rhb1 mRNA levels from control and strains carrying the indicated ectopic alleles, incubated for 8 hours in YP (yeast-peptone) or in nitrate minimal medium amended with glucose (repressive condition) or arabinose (permissive condition). Values are referred to the expression of rhb1 in FB1 (wt strain) grown in YPD. Each column represents the mean value of three independent biological replicates. Error bars represent the SD. (C) The quality of the nitrogen source influences the growth of strains expressing an overactivated Rheb. Serial tenfold dilutions of cultures from the indicated strains (control: FB1 Pcrg1:rhb1), spotted in solid yeast peptone medium or minimal nitrate medium amended either with arabinose (YPA or MMA, permissive conditions for Pcrg1 expression), or glucose (YPD or MMD, restrictive conditions for Pcrg1 expression) as carbon source. Plates were incubated for 3 days at 28°C. (TIF) [file pgen.1010483.s010.tif]

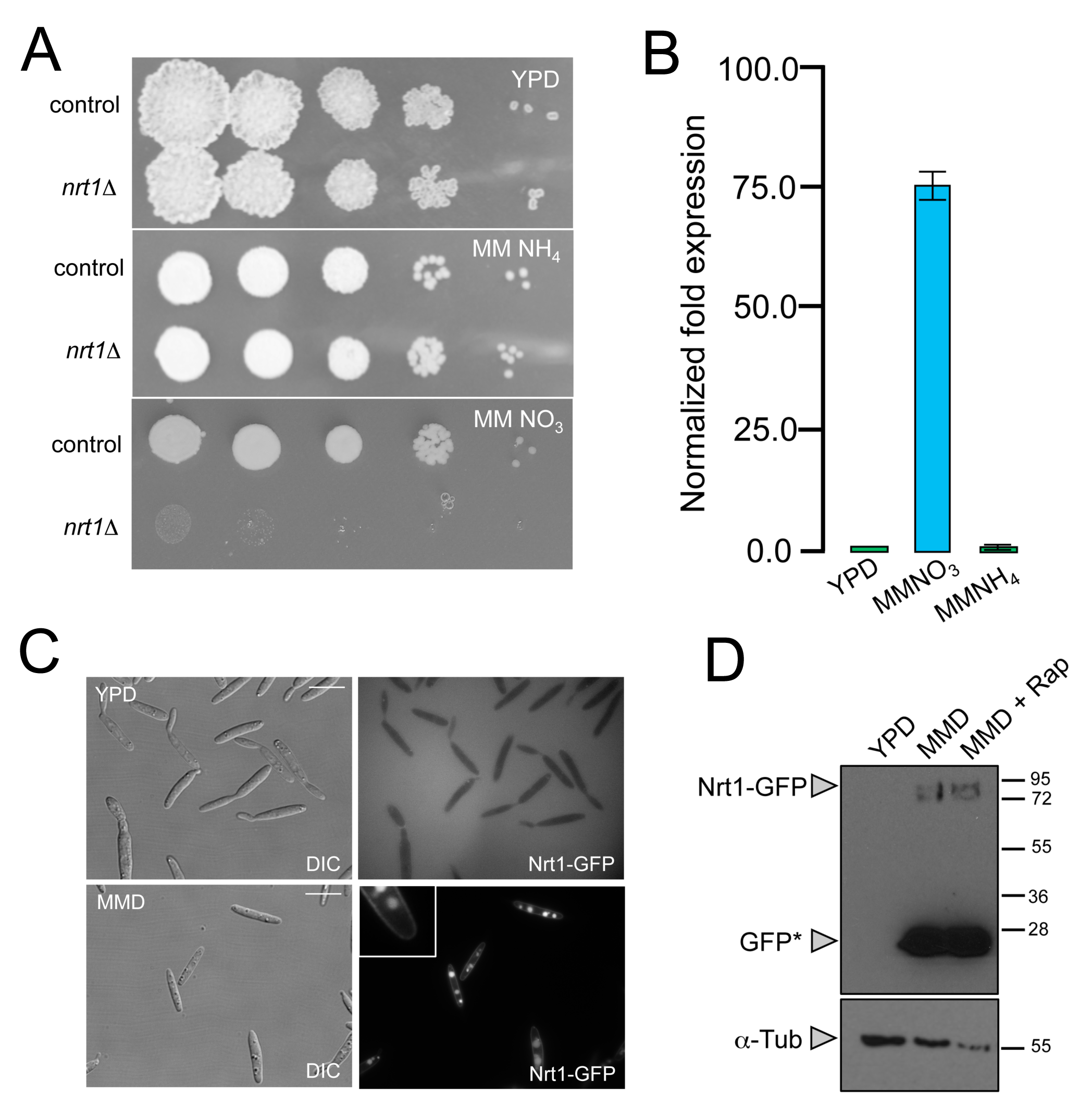

Supplement: S11 Fig — (A) Cells lacking nrt1 were unable to grow in a minimal medium using nitrate as the sole nitrogen source. Serial tenfold dilution of cultures from the indicated strains (control: FB1), spotted in solid YPD, ammonium minimal medium and nitrate minimal medium. Plates were incubated for 3 (YPD) and 4 (minimal medium) days at 28°. (B) the expression of nrt1 was explicitly induced by the presence of nitrate in the medium. qRT-PCR of nrt1 mRNA levels from FB1 cells incubated for 8 hours in the indicated liquid medium. Values are referred to the expression of nrt1 with respect the expression of tub1 (encoding Tubulin α) in the respective growth medium. Each column represents the mean value of three independent biological replicates. Error bars represent the SD. (C) Nrt1-GFP fusion was located at PM depending on the presence of nitrate as the nitrogen source. Fluorescence images of cultures from a strain carrying a nrt1-GFP endogenous allele. Cells were grown for 8 hours in YPD or minimal nitrate medium. A magnification to show the plasmatic membrane accumulation of the fluorescence can be seen in the inset. Images from YPD medium were exposed 6 times more with respect images from minimal medium. Bar: 10 μm. (D) Western blot analysis of cell extracts with anti-GFP antibodies from a strain carrying a nrt1-GFP endogenous allele grown in different media for 8 hours. Levels of Tub1 were used as loading control (bottom blot). In addition to a band corresponding to the expected size of the Nrt1-GFP fusion, we had found a strong signal corresponding to the size of GFP alone. This signal can be explained by assuming that the Nrt1-GFP fusion protein was hydrolyzed in the vacuole (the GFP half is not sensitive to the proteases resident in vacuoles), which is compatible with the proposed mechanism of permeases recycling using the endocytic/vacuole pathway observed in other fungi. Addition of rapamycin (Rap, 1μg/ml) does not alter this pattern. (TIF) [file pgen.1010483.s011.tif]

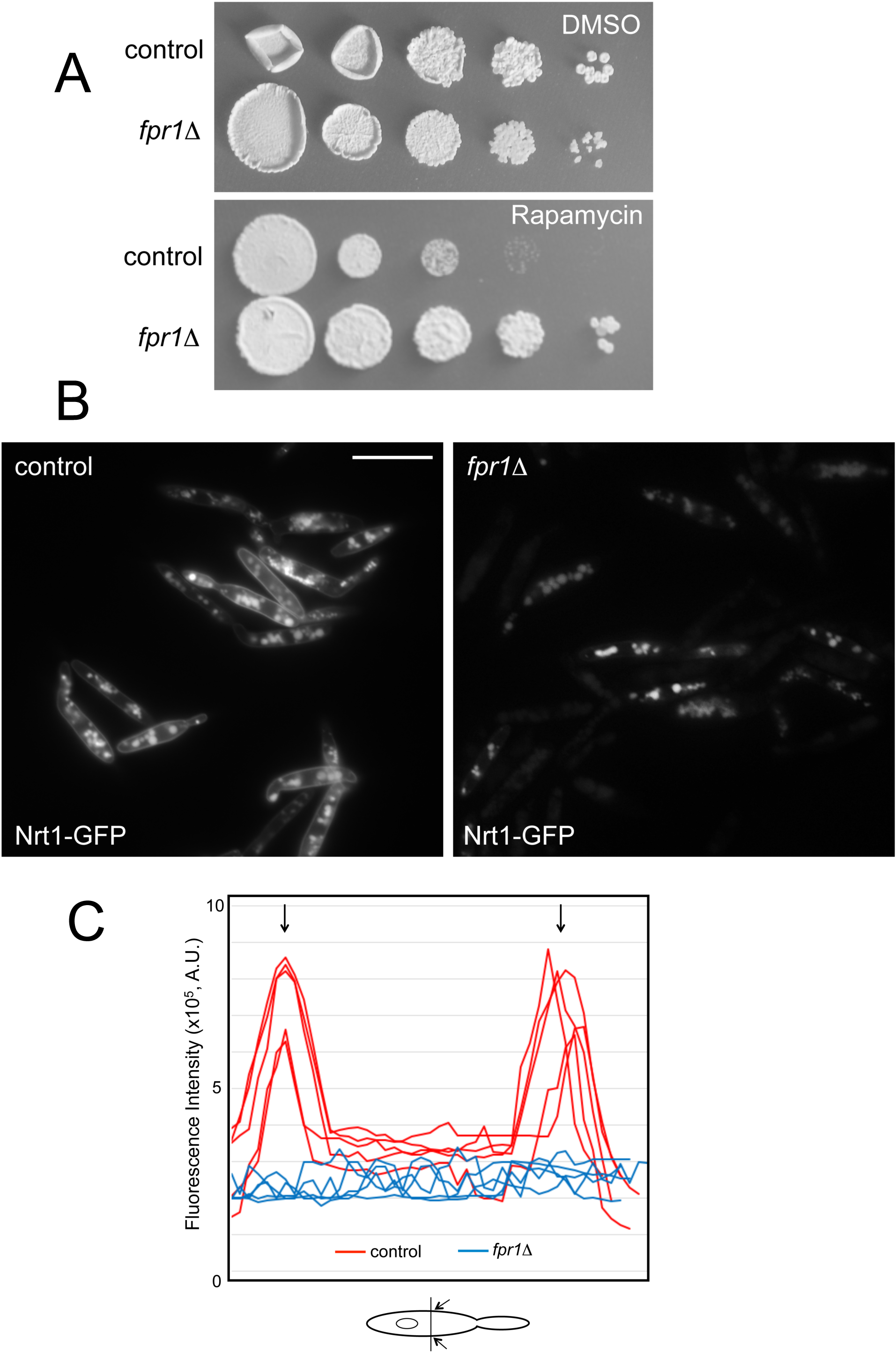

Supplement: S12 Fig — (A) Serial tenfold dilutions of cultures from FB1 (control) and a strain lacking fpr1 (UMAG_11054, encoding the FKBP12 protein) spotted in YPD amended with 1μg/ml rapamycin. Plates were incubated at 28°C for 2 days (control) and 3 days (rapamycin). Control plates included DMSO (1% final) as solvent. (B) Fluorescence images of cultures from the indicated strains (control: FB1 Pnar1: rhb1K127R nrt1-GFP; fpr1Δ: FB1 Pnar1: rhb1K127R nrt1-GFP fpr1Δ) grown for 8 hours in a minimal nitrate medium amended with 1μg/ml rapamycin. Bar: 20 μm. (C) Line-scan analysis of Nrt1-GFP signal intensities from B. 5 cells from each strain were scanned through its medial zone. Arrows indicated the location corresponding to the plasma membrane. A. U., arbitrary units. (TIF) [file pgen.1010483.s012.tif]

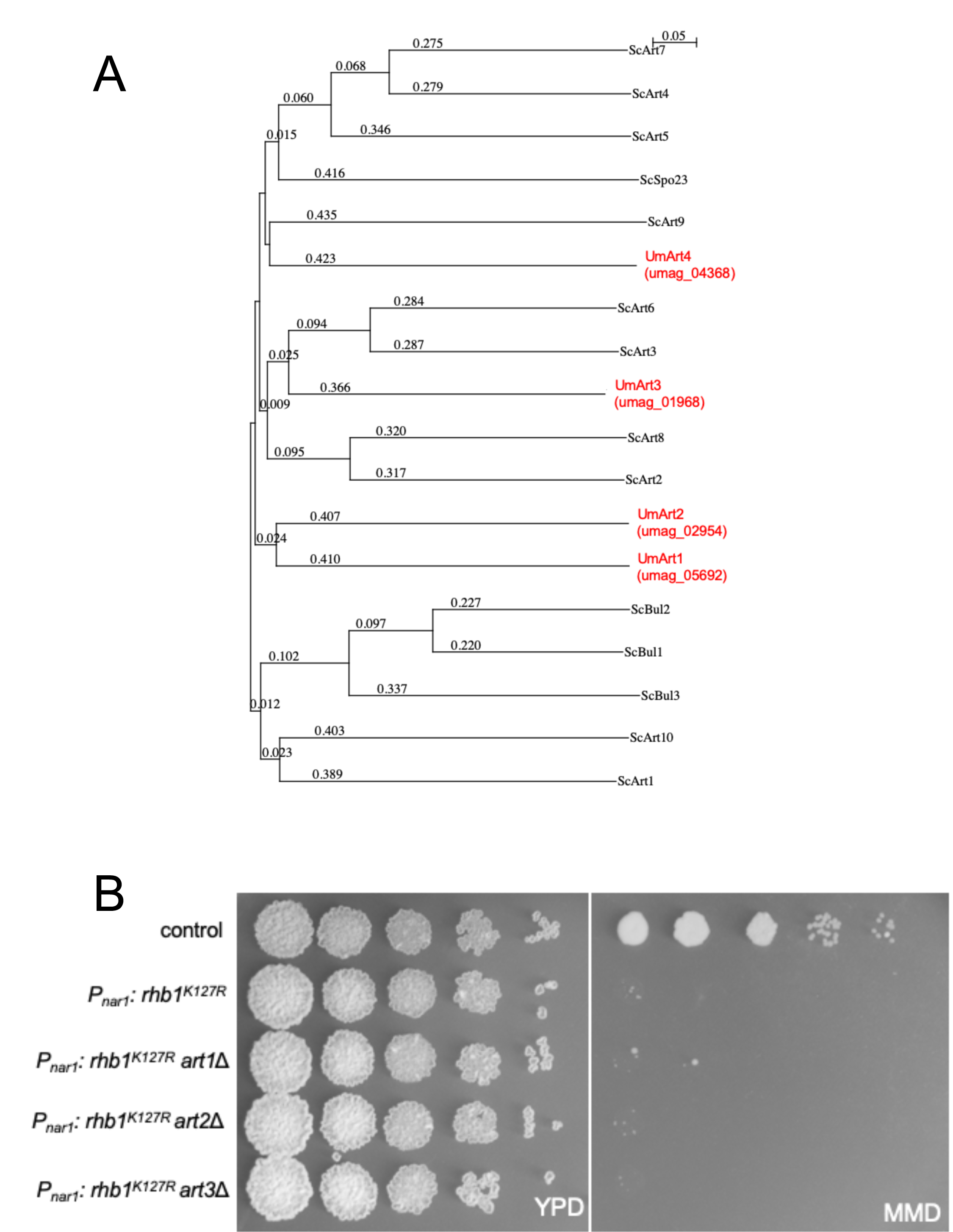

Supplement: S13 Fig — (A) The cladogram was created by a neighbor-joining analysis without distance corrections using ClustalW. The scale bar denotes substitutions per site. (B) Serial tenfold dilutions of cultures from the indicated strains (control: FB1 Pnar1:rhb1), spotted in solid yeast-peptone medium (YPD, restrictive conditions for Pnar1 expression) or solid nitrate minimal medium (MMD, permissive conditions for Pnar1 expression). Plates were incubated for 3 days at 28°C. (TIF) [file pgen.1010483.s013.tif]

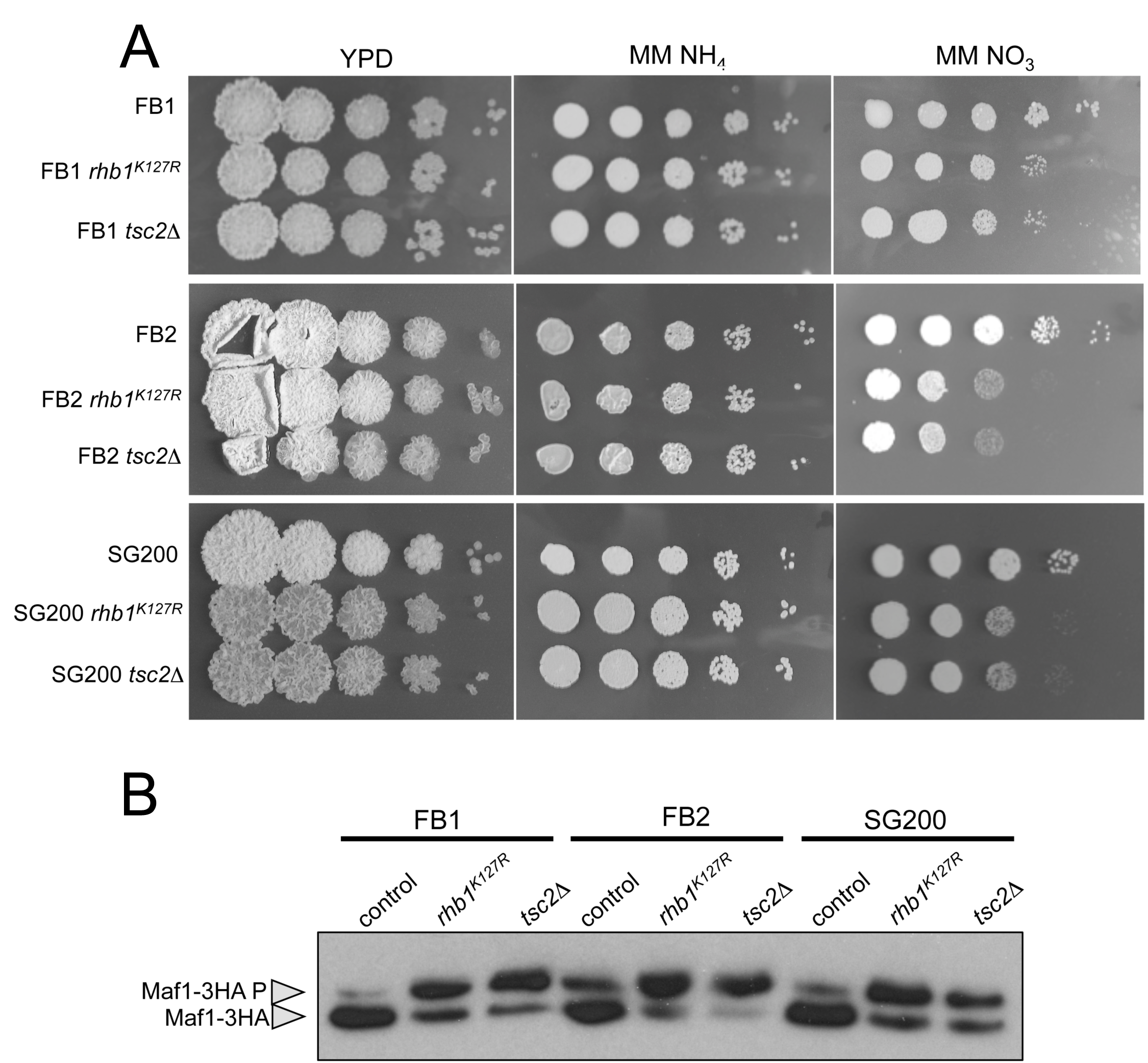

Supplement: S14 Fig — (A) Cells from FB1- FB2- and SG200-derived strains carrying endogenous alleles that up-regulated Rheb1 were spotted in serial tenfold dilutions in YPD, ammonium minimal medium and nitrate minimal medium. The plates were incubated at 28°C for 3 days. (B) Analysis of TORC1 activity using the electrophoretic mobility of Maf1-3HA as readout. Indicated strains were grown for 8 hours in ammonium minimal medium and anti-HA immunoprecipitates from cell extracts were separated in Phos-tag gels and subjected to immunoblot analysis with anti-HA. (TIF) [file pgen.1010483.s014.tif]

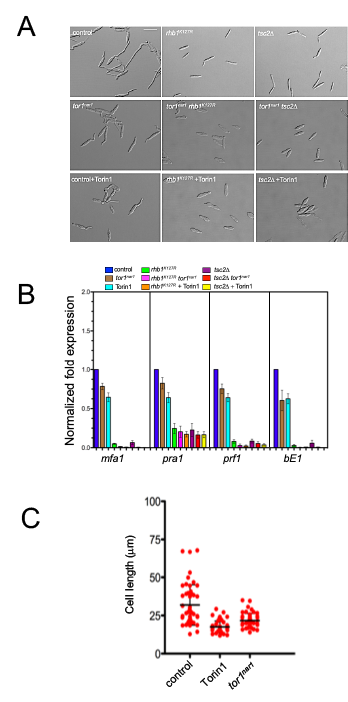

Supplement: S15 Fig — (A) Cell images from cultures of strains carrying the indicated mutations (control: FB1) showing the absence or presence of conjugation tubes upon incubation during 6 hours in minimal ammonium media in presence of synthetic a2 pheromone (2.5 μg/ml). Torin1 was added to 10μM. Bar: 20 μm. (B) qRT-PCR for the indicated genes from control (FB1) and mutant cells incubated for 6 hours in minimal ammonium media in presence of synthetic a2 pheromone (2.5 μg/ml). Torin1 was added to 10μM. Values are referred to the expression of each gene in control strain. Each column represents the mean value of three independent biological replicates. Error bars represent the SD. (C) Tor1 kinase seems to be required for appropriated growth of the conjugation filaments. Control (FB1), torin1 (FB1 incubated 10μM torin1) and tor1nar1(FB1 tor1nar1) cells were incubated during 6 hours in minimal ammonium media in presence of synthetic a2 pheromone (2.5 μg/ml). The length from 50 cells (from 2 independent experiments) was measured and plotted. (TIF) [file pgen.1010483.s015.tif]

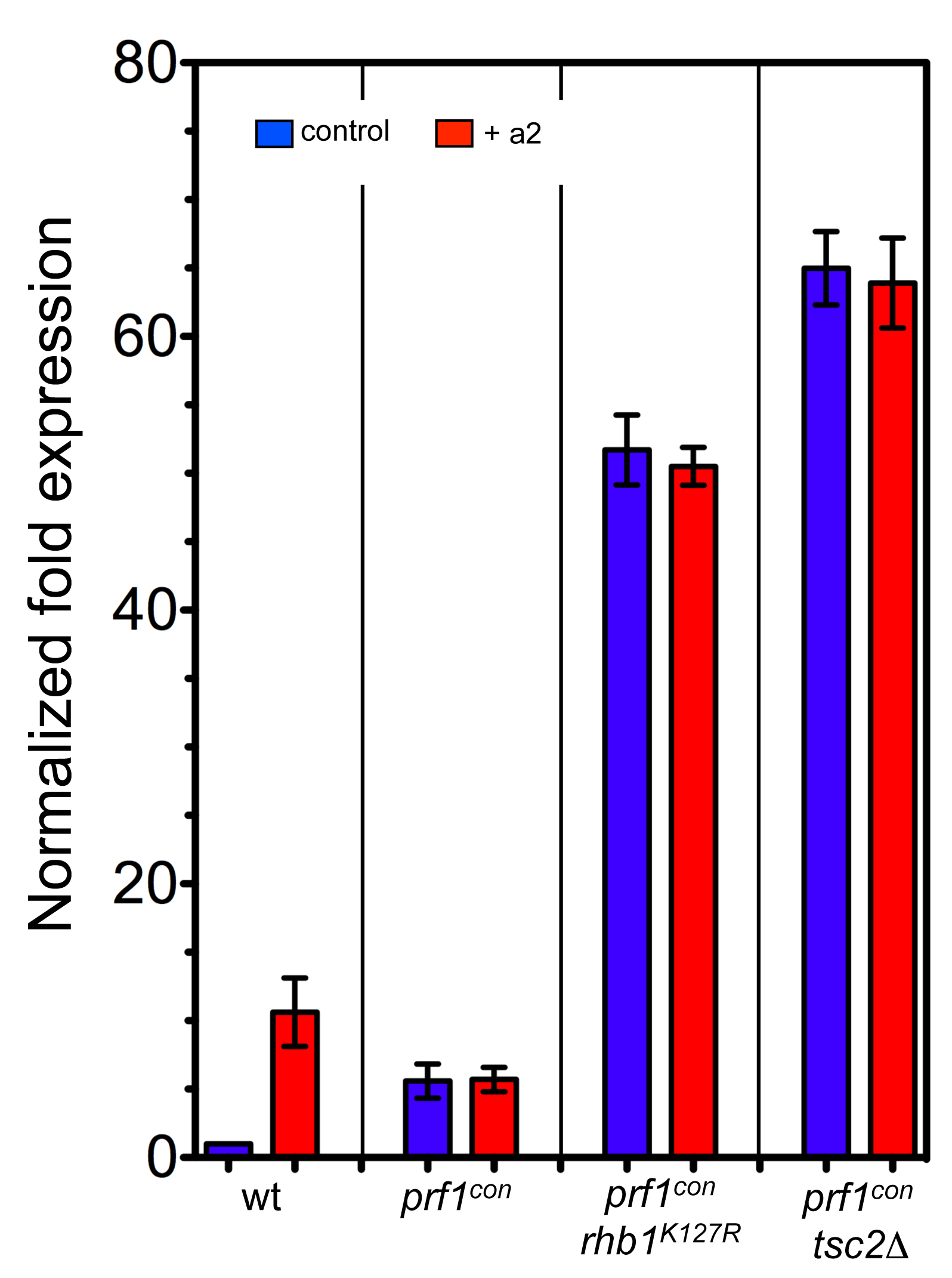

Supplement: S16 Fig — qRT-PCR of prf1 mRNA levels from control (wt, FB1 cells) and strains carrying the indicated alleles, incubated for 6 hours in minimal ammonium media in presence of synthetic a2 pheromone (2.5 μg/ml). Values are referred to the expression of prf1 in FB1 (wt) without pheromone. Each column represents the mean value of three independent biological replicates. Error bars represent the SD. The promoter from tef1 (encoding the translation elongation factor 1), which directs the expression of prf1 in the prf1con allele, is positively activated by TORC1 pathway, explaining the much higher expression of prf1 in the presence of Rheb-activating mutations. (TIF) [file pgen.1010483.s016.tif]

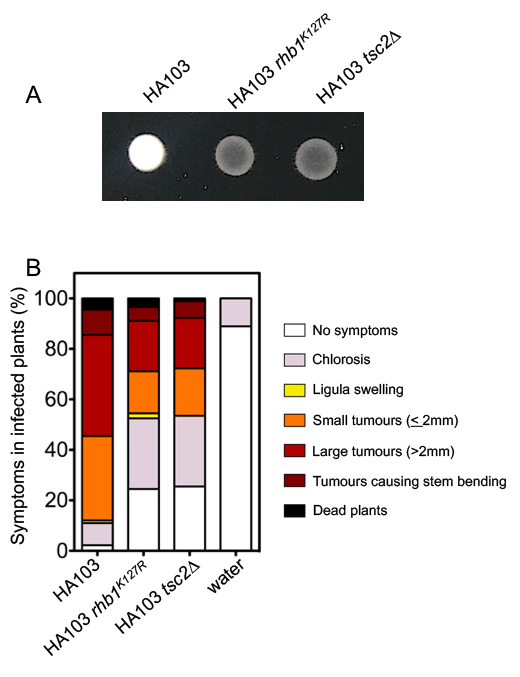

Supplement: S17 Fig — (A) Strains derived from HA103 (expressing constitutively an active b heterodimer) and carrying the Rheb-activating alleles were spotted on minimal ammonium media charcoal plates and incubated for 24 h at 28°C. The white fuzzy colonies reflect the formation of b-dependent filaments. (B) Graph showing disease symptoms caused by infection with the indicated mutant strains. The symptoms were scored 14 days after infection. Three independent experiments were carried out, and the average values are expressed as a percentage of the total number of infected plants (n: 30 plants in each experiment). (TIF) [file pgen.1010483.s017.tif]

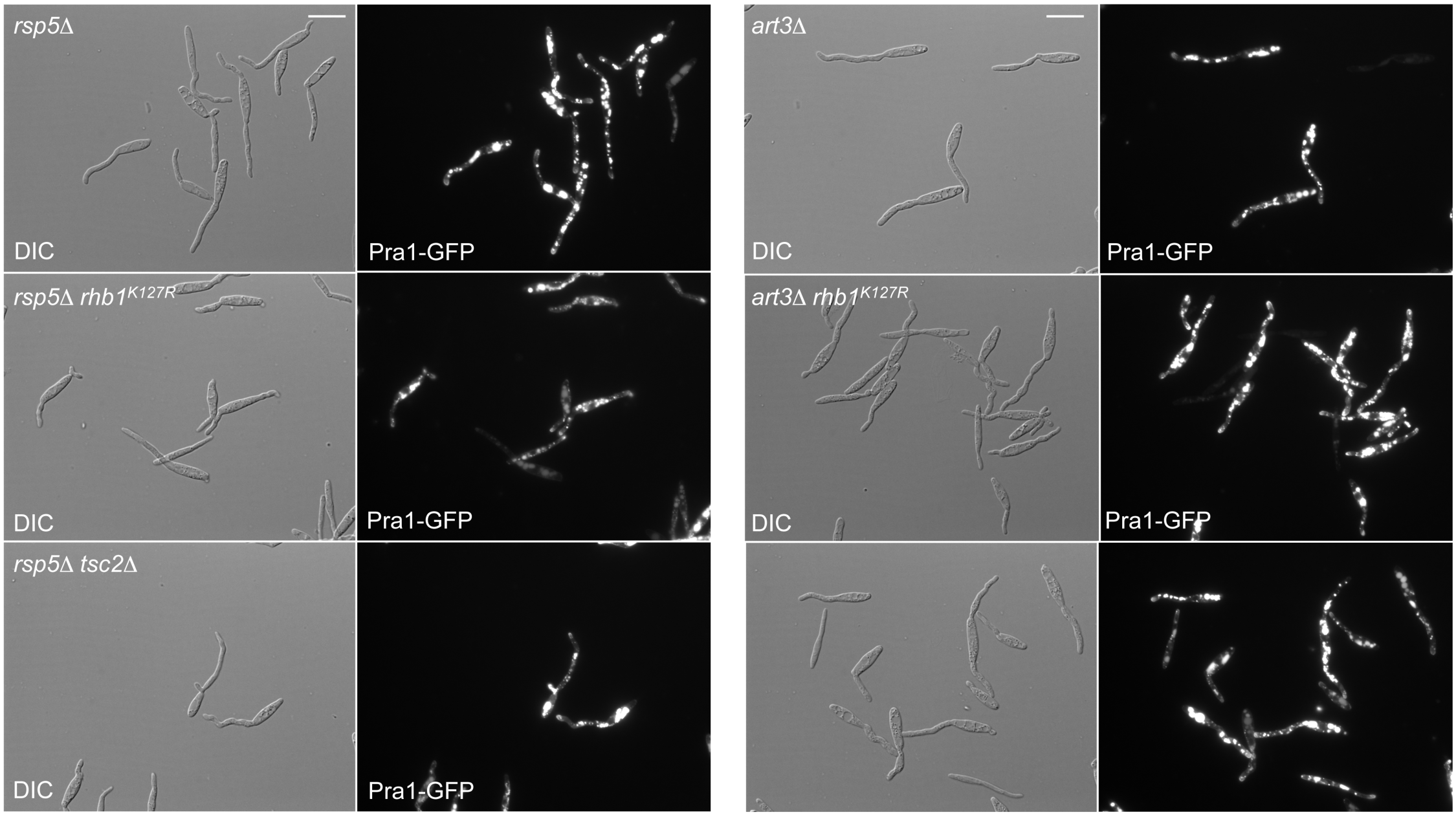

Supplement: S18 Fig — Bar: 15 μm. (TIF) [file pgen.1010483.s018.tif]

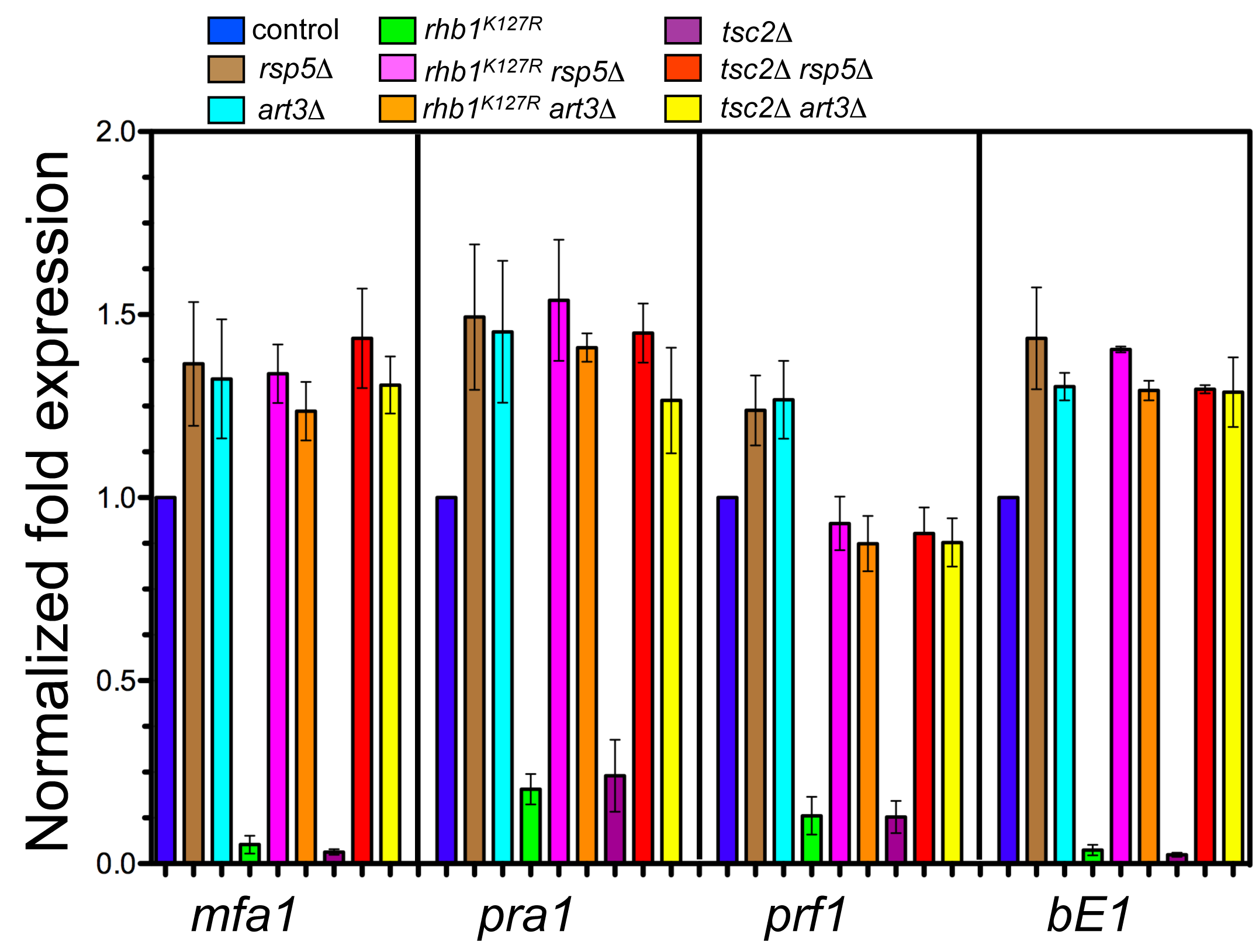

Supplement: S19 Fig — qRT-PCR for the indicated strains (control, FB1 cells) incubated for 6 hours in minimal ammonium media in presence of synthetic a2 pheromone (2.5 μg/ml). Values are referred to the expression of each gene in control strain. Each column represents the mean value of three independent biological replicates. Error bars represent the SD. (TIF) [file pgen.1010483.s019.tif]

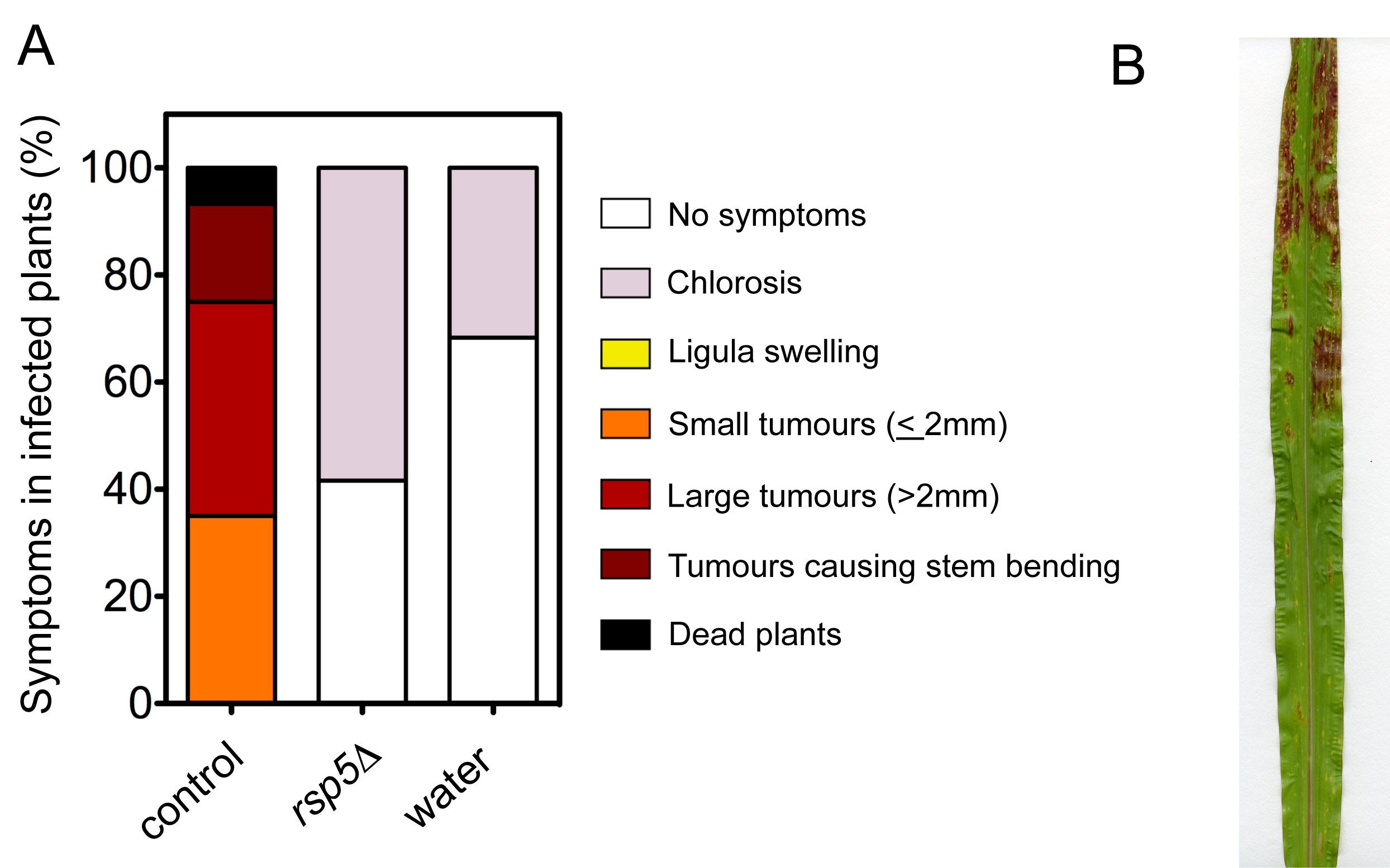

Supplement: S20 Fig — (A) Graph showing disease symptoms caused by crosses of wild-type and mutant strains. The symptoms were scored 14 days after infection. Three independent experiments were carried out and the average values are expressed as percentage of the total number of infected plants (n: 30 plants in each experiment). (B) Representative leave 14 days after infection with the rsp5Δ strains. (TIF) [file pgen.1010483.s020.tif]

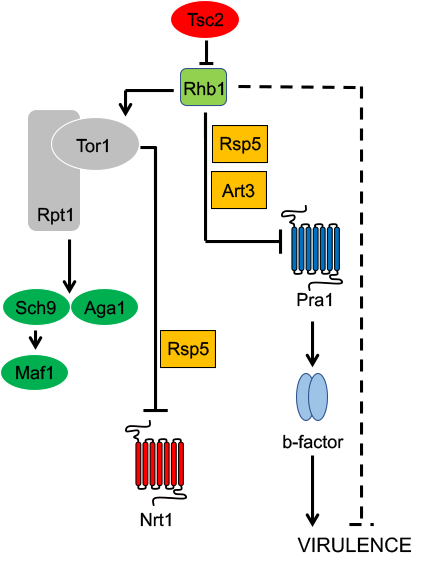

Supplement: S21 Fig — (TIF) [file pgen.1010483.s021.tif]
