# Supplementary material for: The Rheb GTPase promotes pheromone blindness via a TORC1-independent pathway in the phytopathogenic fungus Ustilago maydis
Source: PLoS Genet. 2022 Nov 14;18(11):e1010483. doi: 10.1371/journal.pgen.1010483 (PMC9704768; doi:10.1371/journal.pgen.1010483)
Supplement: S1 Table — (DOCX) [file pgen.1010483.s022.docx]

**S1 Table. List of TOR components from *U. maydis* studied in this work.**

| ***U. maydis*** | **Annotation number** | ***S. cerevisiae***  **(E value)** | ***S. pombe***  **(E value)** |
| --- | --- | --- | --- |
| Tor1 | UMAG_03216 | Tor1 (0)  Tor2 (0) | Tor2 (0)  Tor1 (0) |
| Lst8 | UMAG_03059 | Lst8 (3e^-128^) | Wat1 (2e^-152^) |
| Rpt1 | UMAG_0081 | Kog1 (1e^-127^) | Mip1 (0) |
| Rct1 | UMAG_06215 | Avo3 (1e^-78^) | Ste20 (0) |
| Sin1 | UMAG_00947 | Avo1 (1e^-11^) | Sin1 (5e^-22^) |
| Aga1 | UMAG_10123 | Ypk1 (1e^-171^)  Ypk2 (1e^-168^) | Gad8 (0) |
| Sch9 | UMAG_00602 | Sch9 (5e^-168^) | Sck1 (0)  Sck2 (6e^-137^) |
| Gtr1 | UMAG_11426 | Gtr1 (4e^-74^) | Gtr1 (1e^-78^) |
| Gtr2 | UMAG_12201 | Gtr2 (9e^-8^) | Gtr2 (5e^-12^) |
| Rhb1 | UMAG_05654 | Rhb1 (9e^-49^) | Rhb1 (3e^-82^) |
| Tsc1 | UMAG_10174 |  | Tsc1 (8e^-26^) |
| Tsc2 | UMAG_10623 |  | Tsc2 (1e^-43^) |
