# Supplementary material for: The Rheb GTPase promotes pheromone blindness via a TORC1-independent pathway in the phytopathogenic fungus Ustilago maydis
Source: PLoS Genet. 2022 Nov 14;18(11):e1010483. doi: 10.1371/journal.pgen.1010483 (PMC9704768; doi:10.1371/journal.pgen.1010483)
Supplement: S2 Table — (DOCX) [file pgen.1010483.s023.docx]

**S2 Table. List of strains used in this study**

| **Strain** | **Relevant genotype** | **Source** |
| --- | --- | --- |
| FB1 | *a1, b1* | [1] |
| FB2 | *a2, b2* | [1] |
| SG200 | *a1, mfa2, bW2, bE1* | [2] |
| AUM1 | *a1, b1, tor1^nar1^* | This work |
| AUM7 | *a1, b1, rpt1^nar1^* | This work |
| AUM4 | *a1, b1, sin1*Δ | This work |
| AUM5 | *a1, b1, rct1*Δ | This work |
| AUM36 | *a1, b1, sch9*Δ | This work |
| AUM38 | *a1, b1, aga1*Δ | This work |
| AUM39 | *a1, b1, sch9*Δ, *aga1*Δ | This work |
| AUM26 | *a1, b1, aga1-3HA* | This work |
| AUM32 | *a1, b1, aga1-3HA, rpt1^nar1^* | This work |
| AUM50 | *a1, b1, aga1-3HA, sin1*Δ | This work |
| AUM43 | *a1, b1, maf1-3HA* | This work |
| AUM44 | *a1, b1, maf1-3HA, sch9*Δ | This work |
| AUM45 | *a1, b1, maf1-3HA, aga1*Δ | This work |
| AUM46 | *a1, b1, maf1-3HA, rpt1^nar1^* | This work |
| AUM48 | *a1, b1, maf1-3HA, sin1*Δ | This work |
| AUM80 | *a1, b1, rhb1^crg1^* | This work |
| AUM81 | *a1, b1, rhb1^crg1^, aga1-3HA* | This work |
| AUM82 | *a1, b1, rhb1^crg1^, maf1-3HA* | This work |
| AUM53 | *a1, b1, tsc2*Δ | This work |
| AUM60 | *a1, b1, tsc2*Δ*, aga1-3HA* | This work |
| AUM58 | *a1, b1, tsc2*Δ*, maf1-3HA* | This work |
| AUM84 | *a1, b1, ip::P_nar1_:rhb1* | This work |
| AUM85 | *a1, b1, ip::P_nar1_:rhb1, aga1-3HA* | This work |
| AUM86 | *a1, b1, ip::P_nar1_:rhb1, maf1-3HA* | This work |
| AUM88 | *a1, b1, ip::P_nar1_:rhb1^K127R^* | This work |
| AUM91 | *a1, b1, ip::P_nar1_:rhb1 ^K127R^, aga1-3HA* | This work |
| AUM92 | *a1, b1, ip::P_nar1_:rhb1 ^K127R^, maf1-3HA* | This work |
| AUM154 | *a1, b1, GFP-atg8* | This work |
| AUM153 | *a1, b1, GFP-atg8, tor1^nar1^* | This work |
| AUM155 | *a1, b1, GFP-atg8, rpt1^nar1^* | This work |
| AUM290 | *a1, b1, nrt1*Δ | This work |
| AUM294 | *a1, b1, nrt1-GFP* | This work |
| AUM299 | *a1, b1, nrt1-GFP, ip::P_nar1_:rhb1^K127R^* | This work |
| AUM334 | *a1, b1, nrt1-GFP, rsp5*Δ | This work |
| AUM338 | *a1, b1, nrt1-GFP, rsp5*Δ*, ip::P_nar1_:rhb1^K127R^* | This work |
| AUM300 | *a1, b1, art1*Δ | This work |
| AUM301 | *a1, b1, art2*Δ | This work |
| AUM304 | *a1, b1, art3*Δ | This work |
| AUM310 | *a1, b1, art1*Δ*, ip::P_nar1_:rhb1^K127R^* | This work |
| AUM311 | *a1, b1, art2*Δ*, ip::P_nar1_:rhb1^K127R^* | This work |
| AUM314 | *a1, b1, art3*Δ*, ip::P_nar1_:rhb1^K127R^* | This work |
| AUM368 | *a1, b1, rhb1^K127R^* | This work |
| AUM371 | *a1, b1, rhb1^K127R^, maf1-3HA* | This work |
| AUM42 | *a2, b2, maf1-3HA* | This work |
| AUM369 | *a2, b2, rhb1^K127R^* | This work |
| AUM373 | *a2, b2, rhb1^K127R^, maf1-3HA* | This work |
| AUM65 | *a2, b2, tsc2*Δ | This work |
| AUM70 | *a2, b2, tsc2*Δ,  *maf1-3HA* | This work |
| AUM74 | *a1, mfa2, bW2, bE1, maf1-3HA* | This work |
| AUM370 | *a1, mfa2, bW2, bE1, rhb1^K127R^* | This work |
| AUM374 | *a1, mfa2, bW2, bE1, rhb1^K127R^, maf1-3HA* | This work |
| AUM69 | *a1, mfa2, bW2, bE1, tsc2*Δ | This work |
| AUM76 | *a1, mfa2, bW2, bE1, tsc2*Δ*, maf1-3HA* | This work |
| AUM379 | *a1, b1, rhb1^K127R^ tor1^nar1^* | This work |
| AUM380 | *a1, b1, tsc2*Δ  *tor1^nar1^* | This work |
| HA271 | *a1, b1, prf1^con^* | [3] |
| AUM382 | *a1, b1, prf1^con^, rhb1^K127R^* | This work |
| AUM386 | *a1, b1, prf1^con^, tsc2*Δ | This work |
| FB1Fuz7DD | *a1, b1, ip::P_crg1_:fuz7^DD^* | [4] |
| AUM503 | *a1, b1, ip::P_crg1_:fuz7^DD^, rhb1^K127R^* | This work |
| AUM505 | *a1, b1, ip::P_crg1_:fuz7^DD^, tsc2*Δ | This work |
| AUM507 | *a1, b1, pra1-GFP* | This work |
| AUM509 | *a1, b1, pra1-GFP, rhb1^K127R^* | This work |
| AUM510 | *a1, b1, pra1-GFP, tsc2*Δ | This work |
| AUM514 | *a1, b1, pra1-GFP, ip::P_crg1_:fuz7^DD^* | This work |
| AUM516 | *a1, b1, pra1-GFP, ip::P_crg1_:fuz7^DD^, rhb1^K127R^* | This work |
| AUM515 | *a1, b1, pra1-GFP, ip::P_crg1_:fuz7^DD^, tsc2*Δ | This work |
| AUM523 | *a1, b1, rsp5*Δ | This work |
| AUM525 | *a1, b1, rsp5*Δ,  *rhb1^K127R^* | This work |
| AUM527 | *a1, b1, rsp5*Δ,  *tsc2*Δ | This work |
| AUM524 | *a2, b2, rsp5*Δ | This work |
| AUM528 | *a2, b2, rsp5*Δ,  *rhb1^K127R^* | This work |
| AUM526 | *a2, b2, rsp5*Δ,  *tsc2*Δ | This work |
| AUM541 | *a1, b1, art3*Δ,  *rhb1^K127R^* | This work |
| AUM544 | *a1, b1, art3*Δ,  *tsc2*Δ | This work |
| AUM551 | *a2, b2, art3*Δ | This work |
| AUM552 | *a2, b2, art3*Δ,  *rhb1^K127R^* | This work |
| AUM553 | *a2, b2, art3*Δ,  *tsc2*Δ | This work |
| AUM530 | *a1, b1, rsp5*Δ, *pra1-GFP* | This work |
| AUM532 | *a1, b1, rsp5*Δ,  *rhb1^K127R^, pra1-GFP* | This work |
| AUM531 | *a1, b1, rsp5*Δ,  *tsc2*Δ,  *pra1-GFP* | This work |
| AUM555 | *a1, b1, art3*Δ,  *pra1-GFP* | This work |
| AUM556 | *a1, b1, art3*Δ,  *rhb1^K127R^, pra1-GFP* | This work |
| AUM557 | *a1, b1, art3*Δ,  *tsc2*Δ, *pra1-GFP* | This work |
| AUM601 | *a1, b1, fpr1*Δ | This work |
| AUM602 | *a1, b1, fpr1*Δ,  *ip::P_nar1_:rhb1^K127R^* | This work |
| HA103 | *a1,* P*_hsp70_bW2* P*_otef_bE1* | [5] |
| AUM603 | *a1,* P*_hsp70_bW2* P*_otef_bE1, rhb1^K127R^* | This work |
| AUM604 | *a1,* P*_hsp70_bW2* P*_otef_bE1, tsc2*Δ | This work |

**References**

1. Banuett F, Herskowitz I. Different a alleles of Ustilago maydis are necessary for maintenance of filamentous growth but not for meiosis. Proc Natl Acad Sci U S A. 1989;86(15):5878-82. Epub 1989/08/01. doi: 10.1073/pnas.86.15.5878. PubMed PMID: 16594058; PubMed Central PMCID: PMCPMC297734.

2. Bölker M, Genin S, Lehmler C, Kahmann R. Genetic regulation of mating and dimorphism in Ustilago maydis. Can J Bot. 1995;73:S320-S5.

3. Hartmann HA, Kruger J, Lottspeich F, Kahmann R. Environmental signals controlling sexual development of the corn Smut fungus Ustilago maydis through the transcriptional regulator Prf1. The Plant cell. 1999;11(7):1293-306. Epub 1999/07/13. PubMed PMID: 10402430; PubMed Central PMCID: PMC144278.

4. Muller P, Weinzierl G, Brachmann A, Feldbrugge M, Kahmann R. Mating and pathogenic development of the Smut fungus Ustilago maydis are regulated by one mitogen-activated protein kinase cascade. Eukaryot Cell. 2003;2(6):1187-99. Epub 2003/12/11. PubMed PMID: 14665454; PubMed Central PMCID: PMC326639.

5. Hartmann HA, Kahmann R, Bolker M. The pheromone response factor coordinates filamentous growth and pathogenicity in Ustilago maydis. Embo J. 1996;15(7):1632-41. Epub 1996/04/01. PubMed PMID: 8612587; PubMed Central PMCID: PMC450074.
