# Supplementary material for: The Rheb GTPase promotes pheromone blindness via a TORC1-independent pathway in the phytopathogenic fungus Ustilago maydis
Source: PLoS Genet. 2022 Nov 14;18(11):e1010483. doi: 10.1371/journal.pgen.1010483 (PMC9704768; doi:10.1371/journal.pgen.1010483)
Supplement: S3 Table — (DOCX) [file pgen.1010483.s024.docx]

**S3 Table. List of qRT-PCR primers used in this study**

| primer | sequence |
| --- | --- |
| tor1-a | TTGCTGGACTCAAGAGCAGA |
| tor1-b | GAACGAGGAGAGGCTGTCAC |
| raptor1-a | CGTAACAACAACGGGTCCTT |
| raptor1-b | GCATCGTCTTCCTCTTCAGG |
| rictor1-a | ACGATCTTTGGCTCTCATGC |
| rictor1-b | AGTTTGAACGGTTGGTGAGG |
| sin1-a | GAACGCCTCGATCTTCAAAC |
| sin1-b | CTTGTACGCAGCAGCAATGT |
| tsc2-a | ACACGCCGAGATGGTAATTC |
| tsc2-b | AGGGTGTGCTCTTGAGCTGT |
| sch9-a | ACCTCACCGTCAAAATCGTC |
| sch9-b | GGCCGATGAATTCGTTCTTA |
| aga1-a | CCTCGCTCAGGTCTCTTGAC |
| aga1-b | GTGTTCGGATTGTGTTGTGC |
| rpl43b_a | CGAACTGTCAAGGTCGGAAT |
| rpl43b_b | ACTTGGAGTGCTGCGAGATT |
| s21b-a | ACCAACTTCCCCCTGTCTG |
| s21b-b | CTGGTAGCTCCAGACGTTGC |
| tub1-a | CGAGATGACCTTCTCGTCGT |
| tub1-b | AACATCACCACGGTACAGCA |
| maf1-a | TGGAAAATTCGAGCCTCACT |
| maf1-b | CAAACGTCGACCATAGCAGA |
| rhb1-a | GAGAAAGATTGCCGTTCTCG |
| rhb1-b | GCGTGTTTGGAATTGAGGAT |
| nrt1-a | CTCAAGGCGAAAAGATCGAG |
| nrt1-b | TCAAGCGCTTCAACGTAATG |
| mfa1-a | ATGCTTTCGATCTTCGCTCA |
| mfa1-b | TAGGCAACAACACAGCTGGA |
| pra1-a | GCTCCTGGTCCAGTGTTCAT |
| pra1-b | CAAGACCAAGGCCAAACATT |
| prf1-a | GCTTGCACTCCAGTCACAAA |
| prf1-b | GCTGCATTGGCAGCATAGTA |
| bE1-a | GCTTCTTGGAGTGCCTCAAC |
| bE1-b | GGTGAATTTGCTGGATCGTT |
|  |  |
|  |  |
|  |  |
