# Supplementary material for: The Rheb GTPase promotes pheromone blindness via a TORC1-independent pathway in the phytopathogenic fungus Ustilago maydis
Source: PLoS Genet. 2022 Nov 14;18(11):e1010483. doi: 10.1371/journal.pgen.1010483 (PMC9704768; doi:10.1371/journal.pgen.1010483)
Supplement: S1 Methods — (DOCX) [file pgen.1010483.s025.docx]

**S1 METHODS.**

**Sequence of DNA fragment for Gibson assembly to generate pCas9Rhb1KR (guide template in red):**

5’CAAAATTCCATTCTACAACGCGATTTACATGTGCAGAGACGTTTTAGAGCTAGAAATAGCAAGTTAAAATAAGGCTAGTCCGTTATCAACTTGAAAAAGTGGCACCGAGTCGGTGCTTTTTTTCTAGACCCAGCTTTCTTGTACAAAGTTGGCATTAGTACCCGTACCGAGCTCGAC3’
