# Supplementary material for: The Rheb GTPase promotes pheromone blindness via a TORC1-independent pathway in the phytopathogenic fungus Ustilago maydis
Source: PLoS Genet. 2022 Nov 14;18(11):e1010483. doi: 10.1371/journal.pgen.1010483 (PMC9704768; doi:10.1371/journal.pgen.1010483)
Supplement: S2 Methods — (DOCX) [file pgen.1010483.s026.docx]

**S2 METHODS**

**Sequence of repair template carrying the rhebKR mutation (KR substitution in red, PAM mutation in blue, capital letters indicate coding sequence)**

5’catATGTCTGCTGCTCCGGGAAGCTCGACCCTTCGCAAGAAGAGAAAGATTGCCGTTCTCGGCTCTCGTTCCGTCGgtgagtgacctctcgattagccatccgtcgtcggctttgctgttctcttgcttatcgctctttgccttccgctgcgtcaccaacagGCAAATCGTCACTGATCGTGCGATATGTAGAGGATGCCTTTGTCGACTCCTACTATCCTACGATCGAAAATATTTTCCAGAAAACCATCACCCACAAGGGCCAAGAATACGACTGCGACATTATCGATACGGCAGGCCAGGATGAATACTCGATCCTCAATTCCAAACACGCCATTGGTATTCACGGCTACATGCTCGTCTACAGCATCGCCTCGCGCAATAGCTTCGACATGGTCCAGACCGTATACGACAAGATCTTGAACTACACGGGTACCGAATCGGTGCCCTGCGTGATCGTTGGGCAGCGCTCTGATTTACATGTGCAGAGACAAGTCAGCGAAGCCGAAGGTAAACAGTTAGCAACACAGCTCAAGGCGGCTTGGATCGAGGTCAGCGCAAGACACAATGCCAACGTTGCCAAAGCCTTCGAGGCAATGTTGGGCGAAACGGACAAGGGCACCATTGAGGGCGGTCCTGAACCACAACCTAGCAAGTGCATCGTCATGTAGaattc3’
