# Supplementary material for: The Rheb GTPase promotes pheromone blindness via a TORC1-independent pathway in the phytopathogenic fungus Ustilago maydis
Source: PLoS Genet. 2022 Nov 14;18(11):e1010483. doi: 10.1371/journal.pgen.1010483 (PMC9704768; doi:10.1371/journal.pgen.1010483)
Supplement: Attachment — Submitted filename: Answers Reviewers.pdf [file pgen.1010483.s028.pdf]

Reviewer #1:

In the manuscript by de la Torre and Perez-Martin, the authors investigate the relationship between TOR pathway activity and the virulence program in the corn smut fungus Ustilago maydis. The authors observed that Tor1 inhibition inhibits growth and components of the TORC1 but not TORC2 complexes are essential for growth in U. maydis. The further established Maf1 and Aga1 phosphorylation as potential read-out for TORC1 activity, respectively. To investigate the effects of increased TORC1 activity, the authors express a constitutive active allele of the Rheb GTPase Rhb1 or delete the Rhb1 GAP Tsc2. Rhb1KR expression results in aberrant localization and, most likely degradation of the nitrate transporter Nrt1, which is rescued upon rapamycin treatment, or deletion of the Rsp5 ubiquitin-ligase encoding gene (function downstream of Rhb1). Furthermore, in Rhb1KR expressing strains the virulence program is non-functional and filamentous growth during pheromone perception and formation of the infectious filament in haploid pathogenic background is blocked. Importantly, this phenotype appears to be independent of TORC1 activity as rapamycin treatment did not rescue the pheromone response. As a possible explanation the authors find that localization of the pheromone receptor Pra1 is disturbed upon Rhb1KR expression, which is rescued upon deletion of rsp5 or art3. In case of art3 deletion also rescues, in part the virulence defect of Rhb1KR expressing strains.

In conclusion, this manuscript provides first insight into TOR signaling in U. maydis, but focusses in its main part on the Tor-independent effect of Rhb1 overactivation on the virulence program. The underlying mechanism of Rhb1KR-dependent avirulence might relate to delocalized Pra1 but is not fully explored, understood or experimentally tested. This might also relate to various other reasons, as increased Rhb1 activity results in pleiotropic events.

Overall, the manuscript reports on several interesting observations, but needs to be overworked and restructured to improve coherence and focus. Several findings, appear to be unrelated to the main part of the paper. And based on the

pleiotropic nature of many introduced mutations, conclusions are in some cases speculative.

Specific points:

1. To allow the reader appreciate the central and important findings of the manuscript some parts relating to Figures 1 + 2 + 4 should be more concise and the Rhb1KR mediated avirulence more expanded. Most of the observations in the first sections (page 6-10) conclude with knowledge that is expected and conserved and thus should be clearly shortened. (e.g. that TOR signaling is involved in sensing of nutritional cues)

We agree with the reviewer's observation that most of the data from the first sections conclude with expected knowledge. On our behalf, we have to say that until this work, no reports existed about the description of TORC1 in *U. maydis*. Much of our effort was focused on the definition of ways to disable Tor1 kinase (either by genetic or chemical approaches), so we were able to conclude later on whether our results were dependent on the TORC1 pathway; the description of trustable reporters of TORC1 activity (the Aga1 and Maf1 kinases) and the description of conditions to activate TORC1 at will (the use of Rheb-activating mutations). We considered it essential to describe these previous results (even when some of them were expected) to add solidity to our conclusions about the fact that Rheb acts by using TORC1-independent pathways in *U. maydis*.

2. Please be more concise when introducing the relevant components of the TOR pathway in the individual sections and/or include, when necessary, in the introduction.

We have tried to limit the description of the TOR pathway to the minimum required to understand our experimental approaches. In the first versions of the manuscript, we included all these descriptions in the introduction. However, this resulted in a highly long section. We believe that short introductions to explain the experimental approaches help the better compression of the study.

3. It would be important to find out if Pra1 delocalization is causal for the loss of virulence in Rhb1KR expressing strains. This hypothesis could be

challenged by expression of prf1-con in the haploid pathogenic SG200 or HA103 background (together with Rhb1RK), as this de-necessitates the pheromone response pathway for virulence.

We agree with the reviewer that we only can conclude that there is a correlation between Pra1 delocalization and loss of virulence. We believe that the more direct approach to address this question is to look for mutations on the Pra1 receptor that suppressed the loss of virulence in *rhb1*^{K127R}-expressing strains. We are currently searching for these mutations to understand at which level the unscheduled activation of Rheb would affect the transport of Pra1 to the PM.

Concerning the HA103 strain, we appreciate that the reviewer called our attention to this strain because we have found that constitutive expression of b genes alleviates the negative effect of Rheb-activating mutation on virulence. This result supports our conclusion that the pheromone cascade is one of the targets of unscheduled Rheb signaling. These new results can be found in Supplementary figure 17 and the main text (pages 17 and 23).

4. Please restructure and reword for clarity, grammar, conciseness and correct use of tenses. This is formally a minor point, but based on the numerous occasions these corrections are required to follow the logical flow of experiments.

We asked an English professional editor to check the manuscript. We hope that this new version has fixed all these grammatical mistakes.

5. To me, the title of the paper is somewhat misleading and not fully supported by the experiments. Please rephrase. Based on the data one can conclude that constitutive Rhb1 activity interferes with mating, the pheromone response and formation of infectious filaments. As SG200 is as well affected in virulence and filamentous growth, the Pra1 localization effect is unlikely to be the (only) cause for reduced virulence.

We agree with the reviewer that several causes could be responsible for the affected virulence of Rheb mutants. We believe that at least the interference with the pheromone cascade (or pheromone blindness, as in the new title) is one of

the main problems. The results obtained with HA103 carrying the Rheb-activating alleles indicated that additional problems exist downstream of b function (the level of virulence is lower than control HA103 infections, and the ability to produce *fuz* phenotype in charcoal plates is impaired). However, these defects seem although not to be determinants (the strains are still able to infect the plants). We are trying to figure out at which level Rheb is interfering with the virulence downstream of b function.

The conclusion that if filament formation is reduced in SG200, this shows that there is no defect in cell fusion in compatible matings is not justified (page 15). Rather it appears that both morphological transitions are not functioning.

The reviewer is correct, and we have changed this conclusion (page 15).

Minor points:

1. Please clarify why the effect of Rhb1KR expression on Nrt1 functionality is relevant for the manuscript. In Fig 4E it is referred to RHB1KR-depndent degradation of Nrt1-GFP, although, overall abundance is clearly reduced. As this might as well result from altered translation, the degradation hypothesis is speculative and/or should be tested by further assays (e.g. cycloheximide treatment) or rephrased.

We consider it very relevant to describe that, as it happens in other organisms, *U. maydis* Rheb can act through the TORC1 pathway (as we describe for the Nrt1 permease). This way, we can support that there are both TORC1-dependent and -independent pathways downstream of Rheb.

Concerning the degradation of Nrt1, in Fig4D, in the lane corresponding to Nrt1 levels in the strain expressing *rhb1*^{K127R} (MMD), no band corresponding to Nrt1 is present, but there is a clear band corresponding to GFP. Because it is a protein fusion, there are at least three ways to explain this: vacuolar degradation (GFP half is resistant to vacuolar proteases), alternative transcription start (having a transcription start upstream of GFP ATG), or alternative translation start. From these explanations and considering the studies of transporters in *S. pombe* and

S. cerevisiae, we consider more likely the reason we provide in the manuscript. However, we agree with the reviewer that more focused experimental approaches (like studying degradation kinetics using cycloheximide) could make the conclusion more solid. Because of that, we tempered our findings, and in the text, we consider the degradation of Nrt1 as the most likely explanation, although we cannot discard other reasons.

2. The authors should comment on why their protocols deviate from established U. maydis assays. For instance, precultures for virulence assays are normally done in YEPS medium, pheromone response is normally tested in CM and mating assay on PD charcoal plates. Here the authors use minimal media with ammonium as nitrogen source. It would be important to understand the underlying logic.

The reviewer is right concerning the use of YEPS medium for virulence, CM for pheromone, and PD for charcoal plates. However, a minimal medium with nitrate or ammonium also works fine. Because of the use of the *nar1* promoter to express the *rhb1* mutant alleles, we were forced to use minimal nitrate medium as a growth medium in the first part of the work. Because of that, we considered it more accurate to maintain minimal medium for growth in the rest of the work, so the conclusions about the TOR1C signaling obtained in the first part (such as the activation levels of TORC1 in the Rheb-activating mutants) can be supported. In addition, we found that the growth of cells carrying the endogenous *rhb1*^{K127R} allele or the *tsc2* deletion was affected in PD (we don´t know which nitrogen source is present in this medium) and slightly in CM (probably some of the permeases involved in amino acid transport are affected when TORC1 is highly activated). In contrast, we found that in a minimal medium with ammonium, the presence of Rheb-activating mutations showed no effect in terms of growth. Since we were looking to discard any side effect in development as a cause of defects in virulence, we chose this medium.

3. Formation of conjugation tubes and Fuz7DD filaments should be quantified.

We have included the quantification in the new version of figures.

4. Page 16: The main factor determining Prf1 activity is the differential phosphorylation by Kpp2 and PKA and not the mRNA level, which rather serves as an additional boost for signaling. Please clarify.

The reviewer is correct in this clarification. We rephrased this part to make it clear.

5. Page 17 (also on page 18): The suppression of Rhb1KR effects by Fuz7DD expression is not really convincing, as the morphological alterations upon Fuz7DD expression in WT and Rhb1KR cells are clearly different and do not resemble conjugation tubes. It is hard to see filamentous growth at all.

We agree that the structures produced by Fuz7DD in Rheb-activating mutants are not exactly conjugation tubes. Along the text, we define these structures as something reminding of conjugation tubes. Our impression is that the pheromone receptor has to be polarized to sustain the sharp polar growth characteristic from conjugation tubes. It is known from *S. cerevisiae* that the sustainment of polar growth during shmoo formation requires the pheromone receptor. We want to study in the future whether this is also what happens in *U. maydis*, and we believe that our Rheb-activating mutants could be instrumental in that.

6. I was wondering if the authors could comment on the possibility whether Rhb1KR expression might interfere with cell cycle arrest, as this would explain the majority of the observed phenotypes (absence on conjugation tubes, infectious filaments, virulence).

We analyzed this exciting possibility at the early stages of the work. Because of our background in cell cycle research, we aimed to study the connections between Tor1 and the cell cycle. Much to our disappointment, we never found a case on that (being honest, we started to work with Tor with the expectation that we could see some exciting connection with the cell cycle, and still waiting for it…). Concerning the phenotype we had found on Rheb-activating mutations, they cannot be explained by impairment of the cell cycle arrest. Although cell cycle arrest is essential for the functionality of conjugation tubes, mutants that do not

arrest the cell cycle in response to pheromone can produce conjugation tubes. However, they are multinucleated (see Bardetti 2019 eLife, 8:e48943). In the same way, infective filaments that do not arrest the cell cycle still show polar growth but are multinucleated (Mielnichuk 2009, J. Cell Sci. 122, 4130; Castanheira 2014, Development, 141, 4817). In contrast, cell cycle arrest is mandatory for appressorium functionality (de la Torre, 2021, PNAS 117:30599).

7. In some instances rsp5 is misspelled rps5 in the manuscript.

We fixed it. Thanks for noting!

8. In the discussion it is supposed that TORC1 activity is required to form infective filaments, although no data is presented in this respect. Rather the data indicates that high TORC1 activity might be detrimental for virulence.

We believe that TORC1 could be required for the growth of the conjugation tube. We based this impression on observing the conjugation tubes of wild-type strains grown in pheromone after adding torin1. We have included measurements of the length of the conjugation tubes in Supplementary figure 15C.

Concerning if high TORC1 might be detrimental to virulence, we don´t know. The effects on pheromone signaling seem to be TORC1-independent. However, using the HA103 strain, we have found additional effects of Rheb downstream of b function. We don´t know whether these effects are TORC1-dependent or not (rapamycin does not work on charcoal plates, it probably is sequestered by charcoal). We are currently conducting experiments to address the roles of high Rheb activity downstream of the b factor. And to determine if they require the concourse of Tor1 kinase.

Reviewer #2:

Summary

In frame with previous discoveries, emphasizing the requirement of an obligate inhibition of Tor-signalling for efficient virulence in fungal pathogens, the work conducted by Antonio de la Torre and José Pérez-Martín aimed to further unravel Tor-signalling networks during virulence but also during vegetative growth in the phytopathogen, Ustilago maydis. Because very little is known about Tor-signalling in the vast majority of fungal pathogens, the authors had as prerequisite to assess the existence of TORC components and Tor-signalling, which they remarkably conducted. Besides putting impressive efforts that lead them i) to assess the essentiality of the Tor kinase, ii) to identify experimentally critical components of both TORC1 and TORC2 subunits as well as iii) TORC1 downstream effectors (Sch9 &Aga1 as direct effectors), they also remarkably identified Tsc2 and Rheb as TORC1 upstream regulators and used several genetic approaches, including deletant strains (for Tsc2), conditional alleles and hyperactive mutants, to assess their function as upstream regulators of TORC1. This contrasts with previous studies that mainly addressed Tor-regulation in pathogenic fungi through indirect approaches (i. e. modulating the fungal growth environment). As a major achievement, the authors have elegantly demonstrated that overactivation of Rheb1 hinders poor nitrogen source uptake through internalization and Rsp5-mediated degradation of the nitrate permease Nrt1 in a TORC1-dependent manner. With this in hand, Antonio de la Torre and José Pérez-Martín then focused on their initial problematic (impact of TORC1 overactivation in the pathogenic context) and showed that overactivity of Rheb1 abolishes the ability of U. maydis to form infective structures and thus their ability to infect plants. This was then shown to be due to the pheromone insensitivity, caused by misdistribution of the receptor Pra1, thereby hindering U. maydis to trigger the virulence program. Interestingly, the authors have shown that the inappropriate misdistribution of Pra1 in the virulence context also involves Rsp5 ubiquitin-ligase, as for the TORC1-dependent Ntr1 mis-signalisation previously

demonstrated in this work. Strikingly, the Rheb1-mediated pheromone blindness during virulence was Tor-independent which was unexpected. It is noteworthy that the authors have managed to describe two Rheb1 and Rps5-dependent pathways both regulating external cues modulators of which only one is TORC1-dependent while the other is not.

Significance/originality

The proposed work will be of interest for a broad readership, including the fungi community but also people from the Tor-signalling field, notably because the present work uncovered Tsc-Rheb(-TORC1) dependent pathways that are not or poorly understood even in well-studied organisms. One out of the many strengths of the proposed work is the impressive effort the authors have put to evidence TOR-signalling in Ustilago maydis. It is highly appreciable that the authors dug into the TOR field in filamentous fungi, a field that is dramatically lacking scientific knowledge. It is easily expectable that lessons from this work are transposable to other fungi and will provide important perspectives for future research topics in fungal biology. I would like to point out the fact that the authors have unexpectedly but successfully deleted several genes that were expected to be essential (such as Rct, Sin1, Sch9 or Aga1). The significance of this research obviously also arises from the description of a critical regulation axis for virulence, and thereby evidences potential specific targets for the development of chemicals.

Quality of the manuscript

The manuscript is dense with information and data with very well thought out and carefully planned experiments and controls. The authors have carefully considered alternatives and addressed them with control experiments in nearly all cases. Their conclusions are clearly supported by the data presented. The manuscript is well-written, clear, easy to follow, and the conclusions are well justified. They provide a clear and complete introduction that will be appreciable for readers that are out of the higher fungi biology field. I also appreciated that all along the manuscript, the authors lead the readers by clearly establishing their aims, their issues, how they circumvent these issues and technical details about fungal biology or about the strategies they have employed. This is also

appreciable in the figures within the main manuscript and in the Supplementary Material.

As a general observation, besides the below-mentioned remarks (mainly concerning blots), the figures are of high quality, designed in a sober way which greatly facilitates the reader to catch the author's message. I also appreciated the way the authors have organized the figures between the main manuscript and the supplementary materials.

Remarks

• A main strategy used by the authors was to introduce an ectopic version of Rheb-K127R under nar1 promoter (overexpressed in the presence of nitrate (MMD) but not YPD). My understanding is that the endogenous Rheb might be inhibited on MMD media leading to TORC1 inhibition due to the poor nitrogen source. At the same time, the ectopic Rheb-K127R might be overexpressed leading to active TORC1 which is expected to induce Ntr1 degradation. Consequently, the cell should have defects in nitrate uptake which would lead to a negative feedback loop on the nar1 promoter. Nevertheless, the authors have shown that in spite of this potential negative feedback, the ectopic Rheb-K127R is overexpressed. This is surprising but can be explained due to the presence of alternative nitrate permeases.

My concern here is about the appropriateness of using a nitrate dependent promoter when aiming to unravel mechanistic insight involving nitrate uptake (i.e. via the Ntr1 permease). This point is according to me highly significant, and the authors should provide strong arguments defending why this strategy is still appropriate (or alternatively provide further data to circumvent this issue).

The concern of reviewer is correct. At the early stages of this work, we meditated carefully which promoter use to express the *rhb1*^{K127R} allele. There are 3 regulatable promoters in *U. maydis*: two of them, P*nar1* and P*crg1* are controlled by nutrients (nitrate and arabinose, respectively), while the third one is an artificial

construct controlled by tetracycline. From these, only *nar1* and *crg1* promoters are tightly repressed. We also found that in permissive conditions for each promoter, the level of induction of *nar1* was more similar to the level of expression observed in the endogenous *rhb1* locus: Compare the levels in induction conditions using *nar1* (4 times concerning native promoter, Fig S9) with the use of *crg1* (12 times concerning native promoter, FigS10B). In addition, because the induction has to be done in minimal medium (where the level of TORC1 activity was low, see fig. 2D), to address whether the proposed overactivation by Rheb bypassed the nutritional constraint, we reasoned that using *nar1* would be more convenient, because only the nitrogen source was modified (to use *crg1* in this conditions implies to change the nitrogen source, peptone to nitrate and the carbon source, glucose to arabinose). We never anticipated the side effect on Ntr1. Still, as you pointed out above, even though Nrt1 goes down-regulated, other alternative permeases were enough to at least maintain an intracellular level of nitrate competent to maintain the nar1 expression. Anyway, we also used the *crg1* promoter with a similar result (Fig S10C).

• ***The authors have conducted several immunoprecipitation (IP) experiments and evidenced changes in the phosphorylation status as readouts for TORC1-dependent and -independent pathways. They applied this approach in several cases by taking advantage from band shifts using SDS-PAGE conducted with phostag-gels. Although their results are convincing per se, I was somewhat surprised by the systematic lack of evident controls that must be done when conducting IP experiments. These include a blot showing the inputs that were submitted for IPs.***

The main reason for using IP was to ensure the reproducibility of Phostag gels. We experienced that loading Phostag gels with crude extracts added a degree of uncertainty respecting the reproducibility of the gels (some of them worked, others did not). After consulting some colleagues with more experience than us in Phostag gels, they proposed we use more cleaned samples through IP, which worked very well.

As the reviewer points out, when conducting experiments to detect IP samples, analyzing the IP fraction versus input is essential, especially if the IP procedure

was to investigate the interactions of two proteins, where knowing the input of each protein is very important. In our case, it was simply a “cleaning” step and how much of the input was immunoprecipitated was not that important (although it was highly reproducible) because we analyzed the ratio of phosphorylated versus non-phosphorylated independently in each immunoprecipitated sample.

We made previous IP assays using dynabeads to address how efficient the IP was. As you can observe in Figs S7B and S7C, the HA signal was extremely clean even in crude extracts, and the level of reproducibility of the IP was very high. With this information in hand, we considered unnecessary to analyze each of the samples from each experimental condition. Once we put to work the IP protocol, it was included as a step more in the preparation of samples. We never analyzed for each piece in each experiment how efficient the IP was, confronting input versus IP. We always use the same protein amount in the crude extract used for IP, so at the end of the process we load similar amount of total IP protein in each gel.

• Several blots are overexposed which hinders the evaluation of potential changes in protein amounts or potential double-bands (not applicable for blots evidencing on the same blots distinct bands on a same lane with drastic intensity variations such as free GFP accumulation versus full-length protein tagged with GFP, (for example Fig. 4C). Nevertheless, the authors could in this case cut the blot or display two expositions).

The blot signal was acquired using a chemiluminescence system to help the posterior quantification (we added the quantification in the new figure versions). Because in some bands, the levels were very low (for instance, in figure 2B, the group of phosphorylated Aga1 in rapamycin-treated samples, or Figure 3F, the story of non-phosphorylated Aga1 in Pnar1rhb1 grown in YPD), we decided to have long acquisitions to avoid signal loss in some samples.

• Fig 4C: Loading control is present but no quantification. Because the loading is not homogenous, it is difficult to fully exploit this blot without any quantification. This is even more true because of that the loading control signal is somewhat saturated. Especially, I am pointing this out

because the protein amount of Rhb1 wt + rps5 deletant strain on MMD (control lane 4) is clearly lower than the 3 other control conditions (when comparing Tubulin amounts). Thus, it is expectable that Nrt1-GFP level is comparable in the Rhb1 wt + rps5 deletant strain (if not higher) to the control strain on MMD. This would even further support the authors' expectations. Quantifications would also facilitate the confrontation between protein (4C) and mRNA (4D) levels.

This gel's main aim was to show that Nrt1 disappeared when Rheb was activated and alleviated upon rapamycin treatment or *rsp5* deletion. Respecting that particular gel, the *rsp5* mutation resulted in very sick cells. Although we loaded a similar amount of protein (measured as protein concentration on the crude extract), we repeatedly observed that tubulin levels were lower in extracts from *rsp5* mutants. Tubulin is often used as a loading control, assuming that the experimental conditions do not affect their intrinsic levels. Still, we suspect that, for some reason, *rsp5* loss-of-function affects the tubulin concentration. In this experiment, we cannot say anything about how efficient the degree of suppression was (which has to imply a more careful quantification, looking for a loading control not affected by our experimental conditions).

• The authors have successfully deleted several genes, including some of them that were expected to be essential. One might question whether these deletant strains have not accumulated suppressor mutations that complement the loss of the targeted gene? This point is not clearly discussed. How would the authors argue to this?

Some of the genes we deleted were expected to be essential. For instance, the TORC2 components, Rictor (Rct1) and Sin1, were not essential. These mutants grew slightly slower (see FigS4B), and the cells were enlarged in liquid cultures (Fig S4A, C). Apart from this, we never noticed the presence of any suppressor (that on solid plates used to appear as a colony of faster growth).

Regarding Sch9, it was a surprise that the deletant was alive (Sch9 is essential in *S. cerevisiae*, for instance), but we never noticed any suppressor. Apart from

the construction of the loss-of-function mutant shown in Fig2A, and our attempts to tag the gene, we do not perform more experiments with this mutant.

We never noticed suppressors for *tsc2* and *rhb1K*^{127R} (endogenous locus) as we maintained the cells in YPD. When culturing for a long time in nitrate minimal medium plates, we saw with low frequency (roughly 10^{-4}) the appearance of colonies with faster growth. For each experiment, we always started from freshly streaked YPD plates.

The arrestin loss-of-function mutants (except for *art4*, which never obtained a deletant) appear normal, and we do not analyze further than YPD and minimal medium.

The only deletant that gave us problems was the deletion of *rsp5*. These cells are very sick, and we frequently observed colonies of fast growth that we assume are second-site suppressors. We always started from single isolates with mutant appearance directly obtained from frozen stocks to avoid the accumulation of these suppressors.

• In their work the authors attempted to demonstrate the essential character of several genes. To assess this, they took advantage of inducible/repressible promoters and convincingly reached their aims. However, the authors have recently successfully developed an Auxin-degron based approach in Ustilago maydis to create conditional null-mutants (through inducible targeted protein degradation; https://doi.org/10.1093/genetics/iyab152). I wondered why they didn't take advantage from this system to assess the essentiality of the targeted genes.

The system we put to work in *U. maydis* was a Cre-dependent system for targeted gene deletion. The interest in constructing this system arises after the work we are trying to publish here, because it allows us to obtain conditional mutants independently of culture media (the Cre was activated upon tamoxifen addition). We constructed the system once a significant part of the current work was done. Now, for future studies (see below), we are using this system.

• Fig1F: Despite the author attest that a similar amount of protein was loaded, the associated loading control is missing and there are evident differences in the Atg8-GFP signal. For example, in the second blot, lane 1 (MMD control) contains lower amount of Atg8-GFP than YPD torNAR. Is this due to differences in sample loading or to a physiological effect? This enables one to speculate that free GFP would also be present in the control strain on MMD if an equal amount was loaded. To avoid such issues, a loading control would be necessary. Also, the signals are saturated, and quantification is missing, even if I totally understand that the aim here is on the qualitative level rather than quantitative.

I am a little confused about this question. On Fig1F, second blot, lane 1 is the control for lanes 2 and 3 (the growth medium is the same in the three first lanes), and for lane 5 (YPDtor1nar), the control is lane 4 (control strain grown in YPD). Anyway, the reviewer is correct, and even though we tried to load a similar amount of protein, the band corresponding to GFP-Atg8 is around twice on intensity in lane 5, respecting lane 4. Because we acquire our images using a chemiluminescence system to help the posterior quantification (one of the reasons it seems overexposed), we can add quantification to each lane (free GFP versus GFP-Atg8), and we added these values to the bottom of the gel (average from three independent experiments).

• Fig 8C: The blot is of low quality but sufficient to understand the authors' message. For more clarity, I would suggest lowering "Pra1-GFP" mention in front of the expected band (67,63 kDa). Do the authors have an idea to what correspond the upper band at 250 kDa? Here I would also suggest to kindly remind the readers that defect in virulence due to variations in Pra1 levels was excluded as shown in figure 7B.

We tried our best with this blot but never obtained much better quality. The reason for that was that the signal corresponding to free GFP was very strong. To detect the signal corresponding to Pra1-GFP, we had to cut the membrane between the position corresponding to the expected size of Pra1GFP (around 67 kDa) and the size corresponding to GFP (about 25 kDa) to be exposed to each half of the membrane at different times (seconds for GFP alone, minutes for Pra1-GFP).

The problem was that just in the middle was the size for our loading control Tub1 (around 50kDa). So, to have all the information from a single gel, we have to incubate the membrane with the antibody against Tub1 to expose the membrane. Once the blot was obtained, we stripped off the membrane, cut it into two parts, and incubated it with the GFP antibody. The second blot, especially for long expositions like Pra1-GFP, resulted in the low-quality reviewer pointed out.

Concerning the upper band, we have no idea what it is. We believe that the signal corresponds to Pra1-GFP (it is not present in wild-type strains without the GFP allele), and we think that it is the pheromone receptor modified by some PMT, something usual in membrane proteins. There is only a previous publication of a western from this allele (Fig6A from Fuchs et al. 2006, Plant Cell 18, 2066), but the gel was limited to the lower band. We have decided to show the entire gel.

• The authors showed that while rapamycin has a mild effect on growth in U. maydis (compared to torin1; Supplementary Figure 3.) it was sufficient to restore all phenotypes observed under Tsc-Rheb-TORC1 overactivation (abolishing Aga1 phosphorylation (Figure 2B), ii) restoring the PM-localisation of Nrt1 in a rhb1-K127R (induced expression on MMD; Fig 4A) as well as iii) restoring its protein level (Figure 4C)). The authors concluded that these pathways are TORC1 dependent. Because of the slight inhibitory effect of Rapamycin on growth, could the authors envisage to delete the proline isomerase FKBP12 homolog in U. maydis? In this context, Rapamycin treatment might not induce the above-mentioned events which would further demonstrate their TORC1-dependencies.

This is an excellent experiment! We carried it out, and it is shown in Fig S12. Thanks for it!

• The authors reported Aga1 as being the closest homolog of YPKs (from S. cerevisiae) and Gad8 (from S. pombe). In contrast to YPK1/2 homologs, which are direct TORC2 targets, S. cerevisiae also encodes an additional YPK homolog (YPK3) which is a substrate of TORC1. Of note, once activated by TORC1, YPK3 phosphorylates Rps6. In their work, the authors showed that Aga1 deletion had only a mild impact on growth, then they

showed it to be regulated by TORC1 and concluded it to be a substrate of TORC1. However, they didn't discuss, nor have they tested, the possibility of Aga1 being a functional homolog of YPK3 rather than of YPK1/2. This arises the possibility that the TORC2 substrate (i. e. the functional homolog of YPK1/2) in U. maydis was not identified.

A cladogram including YPK3 (see below) shows that Aga1 is part of the clade composed of all TORC2 substrates from *S. pombe* and *S. cerevisiae* and it is in a different clade as YPK3, so we do not believe that Aga1 is closer to YPK3 than to Gad8 and YPK1/2. Because of that, we were surprised to find that Aga1 (together with Sch9) was located downstream of TORC1 in U. maydis. Reviewer is correct in the sense that another kinase(s) may be located downstream of TORC2 in *U. maydis*. Still, we believe that they will not be characterized just by looking at the sequence because when we searched in the *U. maydis* genome for kinases showing homology to any of the described kinases downstream of TORC1 or TORC2 in budding and fission yeast (including YPK3), the closest hints (apart from Aga1 and Sch9) are kinases related to PKA (Adr1, Uka1), PKC (Ukc1) and PKB (Ukb1), all of them previously characterized in *U. maydis*.

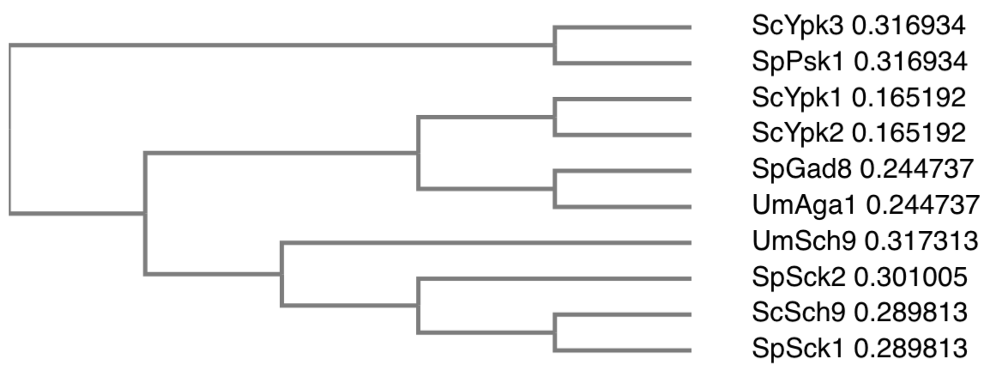

• I would also suggest changing the term S6K by AGC kinases to designate YPK and Sch9 homologs. In facts, S6K kinases are a sub-group of AGC kinases (doi:10.1038/nrm2822 for AGC classification) to which YPK do not belong (they belong to AGC/Akt kinases). Whether Aga1 and other kinases mentioned in the manuscript indeed belong to the Akt or to the S6k is not clear.

The reviewer is correct and we have changed the term S6K to AGC kinases.

• The authors have obtained viable deletant strains of genes expected to encode specific components of TORC2. In spite those genes were not essential in U. maydis, I wondered whether the authors tested the impact of those deletions (i. e. rct and sin1 deletant) for virulence (for example, impact on the development of infective hyphae)? Conversely have they tested the impact of torin1 (inhibiting TORC1 & TORC2 in other systems) treatment during virulence?

We have carried out virulence experiments with *sin1* and *rct1* mutants and found they were avirulent. Currently, we are studying the involvement of TORC2 in virulence, and so far, we have discovered that TORC2 seems not to be required for infective filament formation. However, we have preliminary data that suggest the requirement of TORC2 for appressorium formation.

Concerning the Torin1 impact on virulence, we did not do this experiment, which could be hard to interpret (torin1 will also affect the plant, and in addition is a costly experiment!). We will take advantage of our recently described system to produce fungal conditional mutants inside the plant (the paper in Genetics the reviewer cited above) using the Ce system to remove the distinct scaffold subunits (Raptor, Rictor, and Sin1), and therefore analyze the contribution of TORC1 and TORC2 in different steps of the infection. These approaches are in process now.

• Page 16: There is a little error of inattention while choosing the terms: The authors reported "Elegant previous work had described that Prf1 is submitted to posttranscriptional control by protein phosphorylation… [65, 66]". The herein cited references have indeed evidenced an posttranscriptional regulation of Prf1 but also a posttranslational regulation. I think that the authors meant "posttranslational control" instead of posttranscriptional since they refer to Prf1 phosphorylation.

Reviewer is correct and we rephrased the text.

• Primers table used in the work is missing and should be included

We have included a table of primers in the supplementary section

• ***Suggestion: If I might, although very clearly presented, readers might appreciate a schematic recapitulating both signalling branches (TSC-Rheb TORC1-dependent axis, leading to Rps5 mediated degradation of Ntr1, and TSC-Rheb TORC1-independent overactivation hindering virulence via pheromone receptor Pra1 mis-localisation. In this schematic, TSC-Rheb and rps5 could share the characteristic of being involved in TORC1 dep and indep paths). This schematic could even include the TORC1/(2) upstream and downstream components evidenced here.***

This is a wonderful suggestion. We have included one figure in Supplementary Information (Fig. S21). Thanks!

Reviewer #3

The authors (de la Torre and Pérez-Martin) present an investigation of the role of TORC1 in the virulence of Ustilago maydis using a variety of genetic, pharmacological and biochemical approaches. They made the interesting discovery that a TORC1-independent pathway influences the virulence of the fungus by interfering with the response to pheromone. The main approach was to use a gain-of-function version of the Rheb GTPase, an upstream regulator of TOR, and to assess the roles of TOR-related functions, degradative functions for permeases and receptors, and components of the pheromone signaling pathway. The study is impressive because of the methodical and detailed evaluation of functions related to TORC1 activity, the pursuit of relevant side avenues (e.g., nitrate permease degradation), the use of regulated promoters to bypass essentiality, and the linkage with virulence via infection assays in maize. Overall, the data are clearly presented and interpreted. I have a few minor suggestions for improvements and clarification

1.There are a number of situations where incorrect words are used. For example, the following sentence appears in the Abstract: "Strikingly, this negative control does not imply the TOR pathway." The word imply is not used correctly. Other example words are: concourse, acuation, appropriated, subdued, coherent, seek, etc. I recommend that the authors proof read the entire manuscript to check work usage.

We asked an English professional editor to check the manuscript. We hope that this new version has fixed all these grammatical mistakes.

2.Page 7. Lst8 is briefly mentioned as a candidate gene to be deleted but the authors don't follow up with information about whether the gene was successfully deleted and, if so, whether there was a relevant phenotype.

Lst8 is described as part of both TORC1 and TORC2; therefore, it was less attractive to study than the scaffolding subunits Rct1, Rpt1, and Sin1. Anyway, in the work's early stages, we tried to construct a *lst8* mutant but failed to delete *lst8* in haploid cells. However, we could delete one gene copy in FBD11, a diploid

strain, by inserting a hygromycin resistance cassette. The progeny was analyzed after plant infection with this strain and germination (inducing meiosis) of the diploid spores. No hygromycin colonies were recovered, genetically showing that *lst8* was an essential gene (as expected). No further studies were performed with this gene.

3. Fig. 3F and page 11. More explanation is needed for the statement: "This effect was more apparent in the rhb1K127R allele expression than upon tsc2 removal."

We have removed this statement.

4. It wasn't clear why YPD was used as the restrictive condition for the nar1 promoter – the comparison with MMD as the permissive condition could potentially be confounded by other differences in media composition.

The reviewer is right, the correct repressive condition should be the minimal medium amended with ammonium. However, our long-term experience with the *nar1* promoter in the characterization of cell cycle essential genes taught us that YPD assures a tight negative regulation of the promoter while using a minimal medium amended with ammonium resulted in leaky expression during restrictive conditions. In the cases where we are expressing ectopic *rhb1* alleles, we always compare the ectopic expression of wild-type *rhb1* allele versus *rhb1*^{K127R} to discard any effect unrelated with the specific mutation.

5. Figure 6B,C. Were different concentrations of rapamycin tested to ensure that an appropriate level was employed?

In this experiment, we used the highest rapamycin concentration we could achieve without affecting the morphology of the cells because the solvent (DMSO), which was 1μg/ml. This rapamycin concentration can affect the cells' growth and rescue other TORC1-dependent phenotypes (like the Nrt1 down-regulation). We also analyzed lower amounts (0.5 and 0.1μg/ml) with the same result (no suppression).

6. Figure 8C is quite messy and the GFP bands are highly overexposed. Perhaps the authors can comment on the reliability of their interpretations given the presentation.

The free GFP bands probably are the consequence of vacuole degradation of the Pra1-GFP fusion (the GFP half is not sensitive to vacuolar proteases). It seems that the majority of the Pra1-GFP fusion is submitted to this degradation, while the portion corresponding to full length Pra1-GFP is very minor. To observe the GFP free band, seconds of exposition are enough while the Pra1-GFP requires minutes of exposition. To detect the signal corresponding to Pra1-GFP, we had to cut the membrane between the position corresponding to the expected size of Pra1GFP (around 67 kDa) and the size corresponding to GFP (about 25 kDa) to be exposed to each half of the membrane at different times (seconds for GFP alone, minutes for Pra1-GFP). The problem was that just in the middle was the size for our loading control Tub1 (around 50kDa). So, to have all the information from a single gel, we have to incubate the membrane with the antibody against Tub1 to expose the membrane. Once the blot was obtained, we stripped off the membrane, cut it into two parts, and incubated it with the GFP antibody. The second blot, especially for long expositions like Pra1-GFP, resulted in the low-quality reviewer pointed out. However, we repeated this western at least five times (trying to get good quality), with the same result and conclusion.

7. Given the complexity of the study in terms of the number of genes/mutants/regulated strains employed, the authors might consider adding a summary diagram.

We have done so (Figure S21).
